# Identifying regulatory muscle processes in infantile and adult Pompe patients using a multi‐omic and morphological approach

**DOI:** 10.1111/bpa.70155

**Published:** 2026-10-07

**Authors:** Alexander Schaiter, Keerthika Lohanadan, Alexander Mensch, Andreas Hentschel, Angela Rosenbohm, Cornelia Kornblum, Angela Roth, Tara Procida‐Kowalski, Heidrun H. Krämer, Andreas Hahn, Jörg Höhfeld, Dieter O. Fürst, Werner Stenzel, Peter F. M. van der Ven, Andreas Roos, Marek Bartkuhn, Anne Schänzer

**Affiliations:** ^1^ Institute of Neuropathology Justus‐Liebig‐University Giessen Giessen Germany; ^2^ Department of Molecular Cell Biology, Institute for Cell Biology University of Bonn Bonn Germany; ^3^ Department of Neurology University Medicine Halle Halle (Saale) Germany; ^4^ Leibnitz‐Institut für Analytische Wissenschaften—ISAS—e.V. Dortmund Germany; ^5^ Department of Neurology University Hospital Ulm Ulm Germany; ^6^ Department of Neuromuscular Diseases, Center for Neurology University Hospital Bonn Bonn Germany; ^7^ Institute for Lung Health (ILH) Justus‐Liebig‐University Giessen Giessen Germany; ^8^ Department of Neurology Justus‐Liebig‐University Giessen Giessen Germany; ^9^ Translational Neuroscience Network Giessen (TNNG) Justus‐Liebig‐University Giessen Giessen Germany; ^10^ Department of Pediatric Neurology Justus‐Liebig‐University Giessen Giessen Germany; ^11^ Department of Neuropathology Charité—Universitätsmedizin Berlin, corporate member of Freie Universität Berlin and Humboldt‐Universität zu Berlin, RHC Berlin Germany; ^12^ Department of Pediatric Neurology, Centre for Neuromuscular Disorders University Duisburg‐Essen Essen Germany; ^13^ Brain and Mind Research Institute Children's Hospital of Eastern Ontario Research Institute Ottawa Ontario Canada; ^14^ Department of Neurology, Medical Faculty and University Hospital Düsseldorf Heinrich Heine University Düsseldorf Germany; ^15^ Biomedical Informatics and Systems Medicine Justus‐Liebig‐University Giessen Giessen Germany; ^16^ Present address: Department of Neurosurgery University Medical Center Freiburg Freiburg Germany

**Keywords:** autophagy, glycogen storage, lysosome storage, muscle proteomics, muscle transcriptomic, vacuolar myopathy

## Abstract

Pompe disease is a lysosomal glycogen storage disorder caused by a deficiency of the enzyme acid alpha‐glucosidase (GAA). There are two different clinical phenotypes, which are based on the age of onset, *GAA* variants, and residual enzyme activity. To better understand the regulation of age‐dependent pathways in Pompe disease, we performed a multi‐modal approach. We included mass spectrometry (MS) (liquid chromatography coupled to tandem mass spectrometry) and bulk‐RNA sequencing on skeletal muscle samples from patients with late onset Pompe disease (LOPD) (*n* = 21) and infantile‐onset Pompe disease (IOPD) (*n* = 11) naive to enzyme replacement therapy (ERT), IOPD patients with ERT (*n* = 5) and age‐matched controls. The findings were verified by immunofluorescence studies and ultrastructural analysis using samples with different disease progression. We identified deregulated muscle processes in LOPD and IOPD samples, with different levels of regulation evident at the transcriptome and protein levels. Transcriptomic profiling revealed upregulation of immune processes in both Pompe subtypes, while metabolic processes were downregulated mainly in LOPD. Downregulation of transcripts associated with muscle development and myogenesis was only observed in IOPD samples. MS analysis revealed a decrease in the expression of sarcomeric proteins mainly in LOPD, while chaperone‐assisted selective autophagy was altered in both Pompe subtypes. Proteins linked to vesicle trafficking and biosynthesis correlated with the degree of muscle pathology in LOPD, highlighting their role in the progression of the disease. IOPD patients treated with ERT showed normalised deregulated transcripts, though not fully back to baseline. Proteins increased with ERT were linked with mitochondrial and protein control pathways, and sarcomeric function, indicating ERT's positive effect on skeletal muscle function at the molecular level. Our findings reveal that combining a multi‐omic approach, including morphological observations, enables the detection of a greater number of deregulated muscle processes, indicating both similar and distinct muscle processes in IOPD and LOPD.

## Graphical abstract

The study highlights differences in the regulatory processes of skeletal muscle, such as sarcomeric contractility, lysosomal and autophagic function, metabolic activity and immune regulation, at transcriptomic and proteomic levels in infantile and adult Pompe disease.

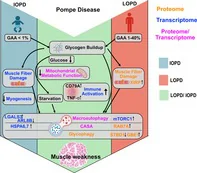

## 1 INTRODUCTION

Pompe disease is a lysosomal glycogen storage disorder caused by pathogenic bi‐allelic variants in *GAA*. The resulting reduced activity of the lysosomal enzyme acid alpha‐glucosidase (GAA) leads to pathological glycogen accumulation in cells of various tissues. Most affected hereby are striated muscle fibres leading to a myopathy, while phenotype severity is dependent on *GAA* variant types and residual enzyme activity of GAA. Clinically, two Pompe phenotypes exist: In patients with infantile onset of Pompe disease (IOPD), GAA residual activity is below 1% (0%–9%) and disease onset before 1 year of age, whereas in patients with late onset of Pompe disease (LOPD) GAA is higher varying from 1% to 40% with disease onset from childhood up to older age [1]. Accordingly, infants present with severe muscle hypotonia and hypertrophic cardiomyopathy and die without treatment because of cardio‐respiratory failure [2]. LOPD patients may develop proximal or axial muscle weakness and respiratory impairment with a higher clinical variability compared to IOPD [3].

Pompe‐related skeletal muscle pathology is characterised by a vacuolar myopathy with lysosomal and extra‐lysosomal glycogen accumulation associated with myofibrillar disturbance and increased autophagy with more severe pathology in IOPD [4]. In LOPD, muscle pathology can range from minimal pathology and subtle glycogen deposits up to a severe myopathy with fat replacement [5].

Pompe disease is the first genetic myopathy in which a causal therapy was established using enzyme replacement therapy (ERT) with recombinant human alpha‐glucosidase [1]. Both IOPD and LOPD patients show a clinical benefit from ERT with reduction of glycogen deposits in skeletal muscle fibres [4, 6, 7]. However, glycogen reduction and cell rescue can be incomplete and Pompe patients may consequently develop a progressive worsening of motor function after several years of treatment [4, 8, 9, 10]. A poorer response to ERT could be a consequence of initiating therapy at an advanced stage of disease progression, limited access of enzyme to the affected cells or disturbed autophagy [11, 12]. The ERT effect on proteomic and transcriptomic level has only been analysed in LOPD so far, using separate approaches whereas the effect of ERT on the molecular level using advanced techniques in IOPD is scarce [13, 14, 15].

The differences in the clinical and morphological phenotypes of Pompe disease may reflect a disease spectrum because of different levels of residual GAA activity. Other factors, such as age‐dependent regulation of muscle processes, may contribute to the disease phenotype [16]. Molecular studies using a multi‐omics approach to decode the molecular processes underlying muscle cell pathology in human skeletal muscle samples of both IOPD and LOPD in one setting have not been performed to date. By analysing skeletal muscle samples for transcriptome and proteome dysregulations in combination with morphological investigations, our study aimed to obtain a deeper understanding of the regulatory processes underlying Pompe disease. Along this line, we performed bulk‐RNA sequencing (RNA‐Seq) and liquid chromatography coupled to tandem mass spectrometry (LC–MS/MS) of skeletal muscle from ERT‐naive Pompe patients with infantile and late onset disease and age‐matched controls which enabled us to investigate the differences of age‐dependent regulatory processes in both Pompe subtypes. In addition, the regulatory effect of ERT was analysed on the molecular level in a smaller cohort of IOPD patients. This study has given us the opportunity to investigate the effect of ERT on the molecular signatures of skeletal muscle in IOPD in one setting.

## 2 MATERIALS AND METHODS

### 2.1 Study design and sample inclusion

We performed comparative comprehensive molecular profiling and morphological characterisation of skeletal muscle from treatment‐naive IOPD and LOPD patients. In addition, a smaller number of muscle biopsies derived from IOPD patients treated with ERT was included. For this multi‐centre study, muscle biopsies were collected from five German Neuromuscular Centers (Giessen, Bonn, Halle, Ulm and Berlin). Inclusion criteria were patients with Pompe disease, with diagnosis based on reduced GAA enzyme activity and two pathogenic variants in the *GAA* gene [17]. Age‐matched control samples were collected from subjects without myopathy according to clinical examination, including laboratory parameters, electrophysiological analysis and absence of any major muscle pathology detectable by thorough light and electron microscopical examination. The control samples were characterised in our previous study [16].

### 2.2 Skeletal muscle pathology

In this retrospective study, all muscle samples had been taken and processed for diagnostic purpose including enzymatic and immunohistochemical staining. For this study, haematotoxin and eosin (H&E) staining was performed on 6 μm thick cryosections. Representative samples were used for Periodic Acid Schiff (PAS) preparations and transmission electron microscopy (TEM). To estimate the degree of muscle pathology from patients with IOPD and LOPD, a morphological score was established on resin‐embedded material in prior studies [4, 5]. In brief, small samples were fixed with 4% glutaraldehyde/0.4 m phosphate‐buffered saline (PBS) and were processed in resin using standard procedures. Semithin sections (1–2 μm) were prepared and stained with PAS and methylene blue. The score was based on the size of glycogen containing vacuoles in addition to large empty vacuoles and disturbed myofibrillar architecture. A score of 0 shows normal morphology, while sections with score 4 represent severe muscle pathology. In our study the score was available from 20/21 LOPD and 5/11 IOPD samples. For TEM images, ultrathin sections (190 nm thickness) were prepared as described before [5]. Sections were examined and photographed using an EM 109 Zeiss transmission electron microscope, equipped with a 2K‐CCD‐Camera (TRS, Dünzelbach, Germany).

### 2.3 Immunohistochemistry

Immunohistochemistry was performed on 6 μm frozen sections using an automated BenchMark XT staining platform (Ventana, Heidelberg, Germany, ultraview universal DAB staining kit) with primary antibodies against MHC I and CD3 (Supporting Information S1: Table 1). Sections were examined using a Nikon eclipse 80*i* microscope.

### 2.4 Immunofluorescence assays

Immunofluorescence staining was performed at 6 μm cryosections. After fixation with acetone (−20°C) for 10 min, sections were air dried, rehydrated with PBS and incubated with blocking solution (10% normal goat serum [NGS] in PBS) for 45 min at 37°C. Sections were incubated with primary antibodies diluted in 1% bovine serum albumin (BSA) in PBS overnight at 4°C (Supporting Information S1: Table 1). Sections were washed twice with PBST (0.5% Tween‐20 in PBS) and once with PBS, and incubated with secondary antibodies (GAM IgA‐Alexa 488 1:200. SBA 1040‐31; GAM IgG1 Alexa 594 1:300, Jackson 115‐585‐205, GAR Alexa 647 1:500, Invitrogen A21245) diluted in 1% BSA in PBS for at least 2 h at 37°C. Sections were washed three times in PBST, followed by a dip in double‐distilled water and mounted with Fluoromount‐G (Invitrogen, Thermo Fisher Scientific, Dreieich, Germany). Microscopic analysis of the sections was performed using an LSM710 or LSM 900 confocal laser scanning microscope (Carl Zeiss GmbH, Oberkochen, Germany).

### 2.5 Molecular and bioinformatic analysis

Twenty 10 μm thick skeletal muscle sections were provided for bulk‐RNA‐Seq and tandem mass spectrometry (MS). To decipher protein changes underlying muscle pathology, in the framework of NMD‐GPS (https://nmd-gps.net/), unbiased proteomic profiling was performed as described previously [18]. Detailed information of molecular and bioinformatic analysis is described in Supporting Information S1 and in a previously published study [16].

In volcano plot, the *x*‐axis displays the log_2_ fold change in expression, and the *y*‐axis displays the negative log10 of the adjusted *p*‐value. Proteins and genes with an adjusted *p*‐value false discovery rate (FDR) of less than 0.05 and an absolute log_2_ fold change of greater than 1 were coloured red. Box plots of differential abundant proteins (DAPs) and differential expressed genes (DEGs) display the median and interquartile range. Outliers were indicated by black dots within the plot. Significance was indicated by the following: *p < 0.05; **p < 0.01; ***p < 0.001; ****p < 0.0001; n.s. = not significant.

Figures 1, 3 and 9 were created with BioRender. Deepl was utilised to verify the manuscript for grammatical and orthographic accuracy.

**FIGURE 1:**
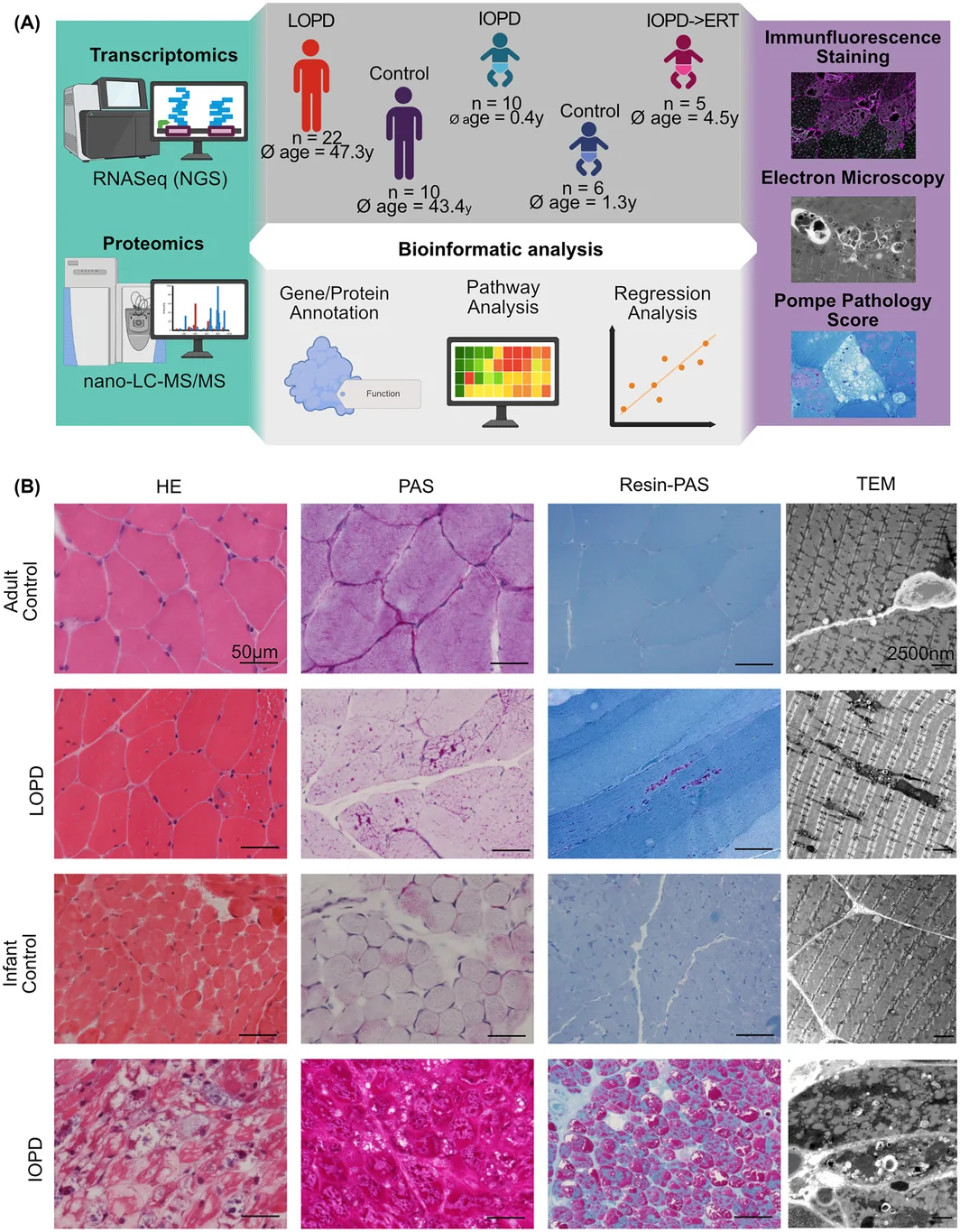
Study work flow and representative skeletal muscle pathology of LOPD and IOPD. Overview of skeletal muscle samples included in the study and applied methods (A). Haematotoxin and eosin (H&E) stained cryosections showed few vacuolated muscle fibres with small glycogen containing vacuoles (PAS) in LOPD presenting minor pathology. The lysosomal and extra‐lysosomal glycogen deposits were more visible in PAS‐stained resin sections and with transmission electron microscopy (TEM). In comparison, in IOPD, the myofibrillar architecture was severely destroyed with loss of sarcomeric structures, accompanied by massive glycogen deposits and large autophagic vacuoles and loss of sarcomeric structures (B).

## 3 RESULTS

### 3.1 Sample characterisation

Muscle biopsies from 36 patients with Pompe disease naive to ERT were included in the study: 21 LOPD patients (aged 31–78 years; mean age 47.4 years) and 11 patients with IOPD (aged 0.1–0.8 years; mean age 0.4 years). In addition, five IOPD patients (2.3–7.3 years; mean age 3.9 years) treated with ERT (20 mg/kg alglucosidase alfa every other week) were analysed. Samples were collected prior to and after ERT treatment from the same patient in two cases. Muscle biopsies from age‐ and gender‐matched healthy individuals of 10 adults (19–65 years; mean age 43.4 years) and 7 infants (0.6–2.3 years; mean age 1.2 years) served as controls (Figure 1A, Table 1) [16]. See Supporting Information S1: Table 2 for details of sample analysis.

**TABLE 1:** Cohort of skeletal muscle samples included in the study.

		LOPD naive	IOPD naive	IOPD ERT treated	Control adult	Control infantile
MS RNA‐Seq	Number	21/21 11/21	10/10 8/10	5/5 3/5	10/10 7/10	6/6 4/6
MS RNA‐Seq	Age at biopsy (years) Mean (range)	47.3 (26–78) 40.9 (36–58)	0.4 (0.1–0.8) 0.4 (0.1–0.6)	3.7 (2.2–7.3) 4.5 (2.8–7.3)	43.4 (19–65) 43.6 (30–56)	1.3 (0.3–2.3) 1.3 (0.6–2.3)
MS RNA‐Seq	Female %	43% 45%	30% 25%	60% 66%	50% 57%	67% 100%
Pathology score	Number Score mean (range)	20/21 0.72 (0.08–2.23)	4/10 2.70 (1.7–3.5)	3/5 2.63 (0.5–4.0)	n.a.	n.a.

Abbreviations: ERT, enzyme replacement therapy; IOPD, infantile‐onset Pompe disease; LOPD, late onset Pompe disease; MS, mass spectrometry; n.a., not analysed; RNA‐Seq, RNA sequencing.

### 3.2 Skeletal muscle pathology in LOPD compared to IOPD samples

All muscle biopsies were reevaluated to ensure that the muscle pathology revealed the distinct Pompe pathology. Cryosections corresponding to the H&E sections were used for both LC–MS/MS and RNA‐Seq analyses. Figure 1B illustrates the muscle pathology of representative samples of ERT‐naive LOPD and IOPD patients and age‐matched controls.

### 3.3 Systematic differences in gene and protein regulation in ERT‐naive Pompe muscles compared to controls

We employed principal component analysis (PCA) to identify overarching patterns and relationships between disease groups and controls. This was achieved by including all transcripts and DAPs with the ‘prcomp’ R function. PCA revealed significant differences in deregulated transcripts and proteins of LOPD and IOPD, each compared to the age‐matched controls. The differences between IOPD and controls were more pronounced than those between LOPD and controls. These findings are consistent with a higher degree of variability in the morphological phenotypes of LOPD patients, including muscle biopsies that present with a less pronounced pathology (Supporting Information S1: Figure 1, Table 1).

### 3.4 Deregulation of muscle transcriptome in LOPD and IOPD compared to age‐matched controls

Using RNA‐Seq analysis, we identified a similar number of expressed genes in LOPD (20.171 variables) and IOPD (20.074 variables). In comparison to the control specimens, one DEG was downregulated and 22 DEGs were upregulated in LOPD. In IOPD, 111 downregulated and 361 upregulated DEGs were identified (both adjusted *p* < 0.05 and log_2_‐FC >1) (Supporting Information S2: Table 3; Figure 2A,B).

**FIGURE 2:**
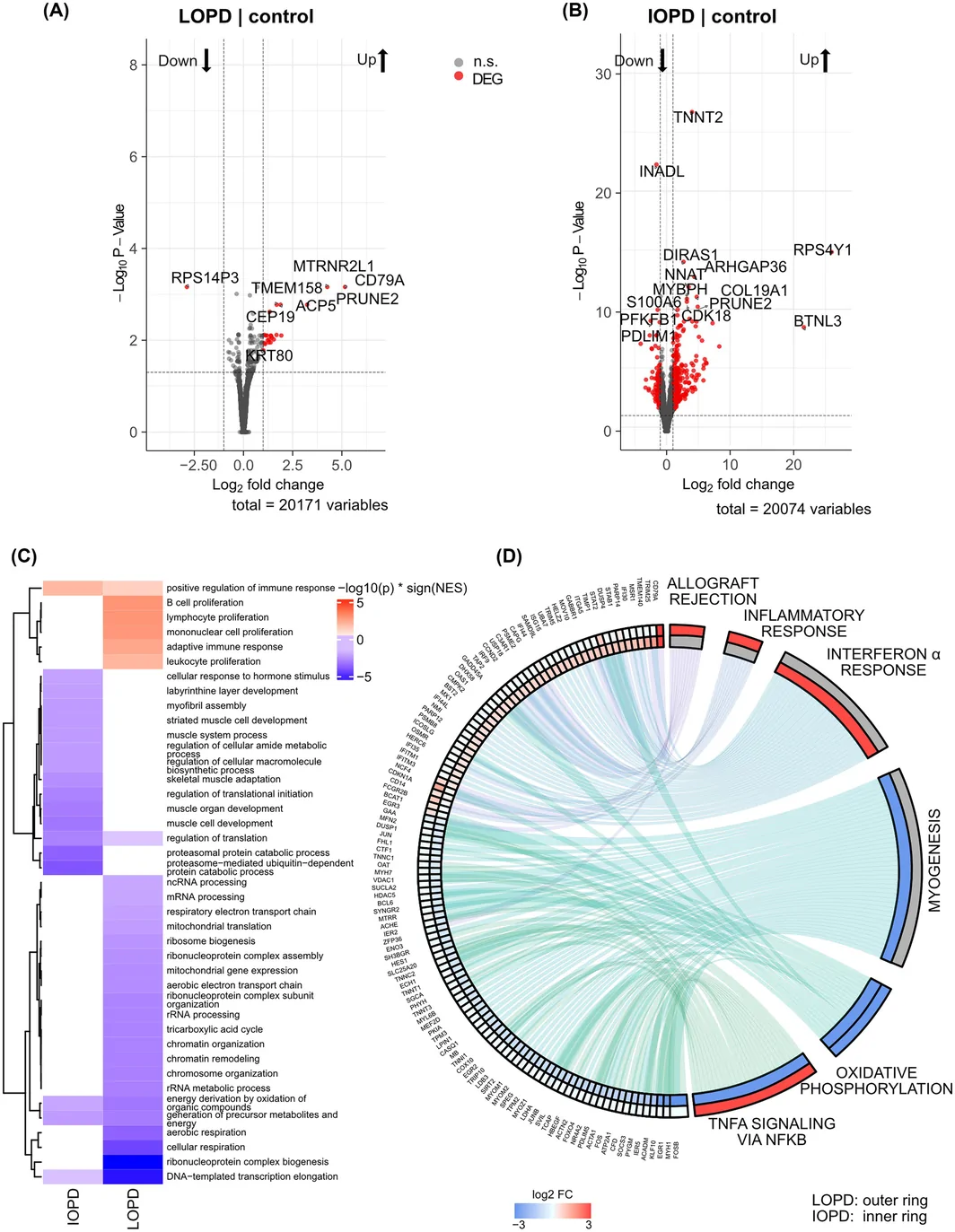
Transcriptomic analysis inLOPD and IOPD compared to age‐matched controls Volcano plot showing 20.171 identified genes with one significantly downregulated and 22 upregulated genes in LOPD (A). Volcano plot showing 20.074 identified genes with 381 significantly downregulated and 111 upregulated genes in IOPD (B). Pathway analysis using GO GSEA (heatmap) and Hallmark (chord diagram) terms revealed differences in selected gene regulation between IOPD and LOPD, when compared to age‐matched controls. Genes associated with myogenesis and development were mainly downregulated in IOPD. Genes associated with immunoregulation were upregulated and genes associated with metabolic processes were downregulated in both, IOPD and LOPD (C, D). DEG, differential expressed gene; NES, normalised enrichment score; n.s., not significant.

Gene ontology (GO) and Hallmark gene sets of IOPD/control and LOPD/control revealed that inflammatory processes such as ‘immune response’ were both upregulated in IOPD and LOPD, whereas ‘myogenesis’ and ‘striated muscle development’ were downregulated only in IOPD. Of note, ‘metabolic processes’ were predominantly downregulated in LOPD (Figure 2C,D). Focusing on the transcripts that have undergone the most significant deregulation revealed that in LOPD, immunoglobulin‐associated transcript *CD79a* and *TMEM158*, which is increased in response to Ras pathway activation, were highly upregulated whereas the ribosomal protein pseudogene *RPS14P3* was profoundly downregulated (Figure 2A). In IOPD, *TNNT2* encoding the thin filament regulatory protein troponin T, *COL19A1* encoding an extracellular matrix protein and *RPS4Y* encoding a small ribosomal subunit were found to be highly upregulated. *INADL*, *PDLIM1* and *PFKFB1* were found to be downregulated (Figure 2B). *INADL* encodes a multiple PDZ domain protein which is involved in protein–protein interactions and the organisation of multimeric complexes at the plasma membrane [19]. The *PDLIM1* gene encodes a cytoplasmic protein that is associated with the cytoskeleton. *PFKFB1* encodes an enzyme that plays a role in glucose homeostasis [20].

### 3.5 Deregulation of muscle proteome in LOPD and IOPD compared to age‐matched controls

MS based protein analytics enabled the robust identification of 2465 proteins of which 830–920 were measured in at least 50% of the samples of one experimental group comparing two experimental groups. Comparing LOPD to matching controls, 830 proteins were identified, of which 41 were downregulated and 27 upregulated in LOPD samples. In IOPD, 920 proteins were identified, of which 10 were downregulated and 16 upregulated (both adjusted *p* < 0.05 and log_2_‐FC >1) (Figure 3A,B).

**FIGURE 3:**
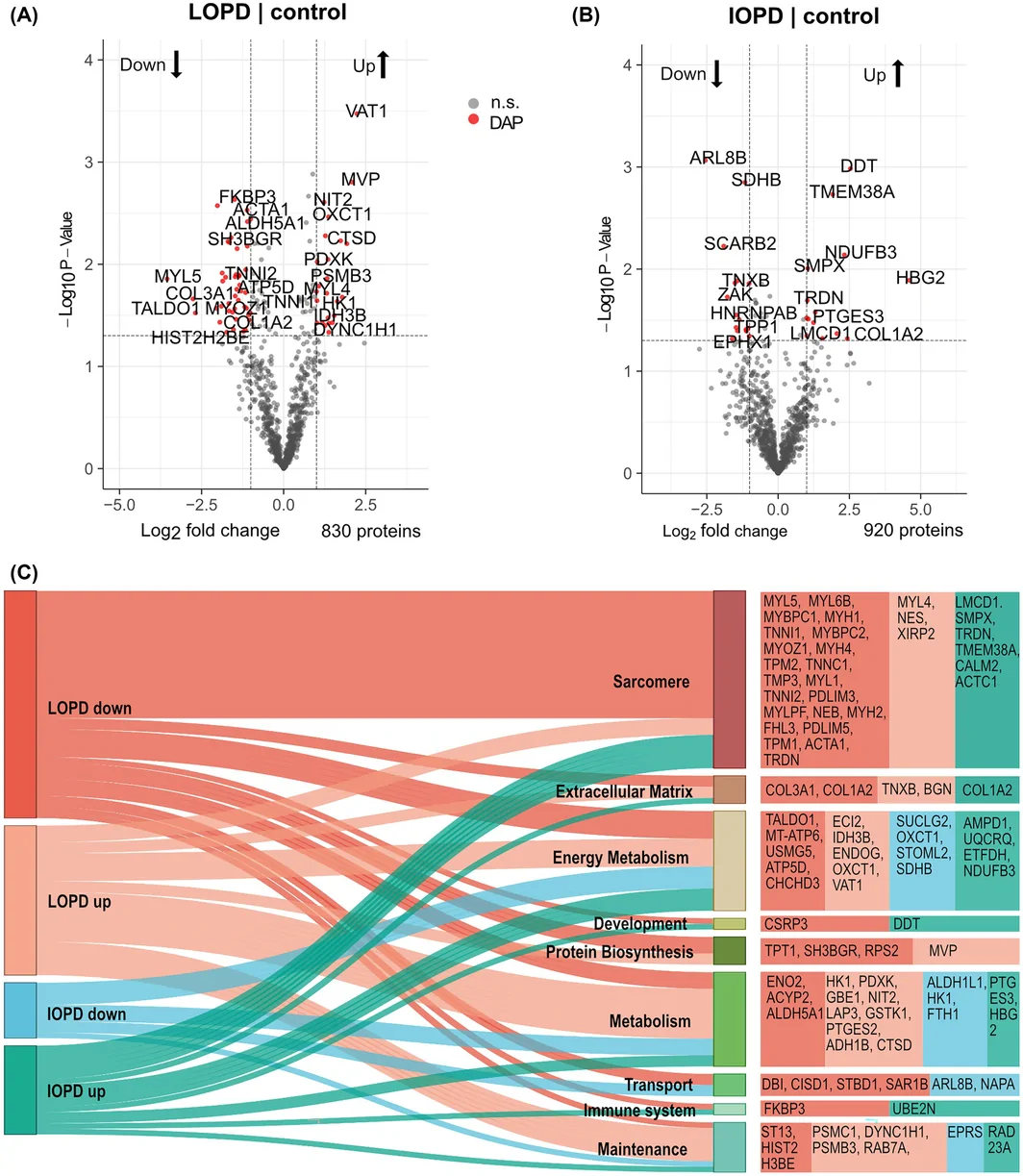
Proteomic analysis in LOPD and IOPD compared to age‐matched controls. Volcano plot showing 830 identified proteins with 41 significantly downregulated and 27 upregulated proteins in LOPD (A). Volcano plot showing 920 identified proteins with 10 significantly downregulated and 16 upregulated proteins in IOPD (B). Sankey diagram illustrating the differences of protein regulation in LOPD and IOPD. In LOPD, the majority of downregulated DAPs belonged to the sarcomere. Proteins associated with metabolic function and muscle maintenance such as autophagic process were upregulated in LOPD. In IOPD, proteins associated with metabolic processes, for example, mitochondrial function, were up‐ or downregulated (C). DAP, different abundant protein; n.s., not significant.

A Sankey diagram was generated to illustrate the differences in protein deregulation between LOPD and IOPD, each group compared to the age‐matched controls. In LOPD, the majority of downregulated DAPs belonged to sarcomere‐associated proteins, while proteins associated with metabolic functions and muscle maintenance, for example, autophagic processes were upregulated. In IOPD, proteins associated with metabolic function, for example, mitochondrial function were either up‐ or downregulated (Figure 3C).

Focusing on the most significantly deregulated proteins in LOPD, vesicle amine transport protein‐1 (VAT1) was identified as the most abundant. VAT1 has been implicated in multiple regulatory processes and may be involved in the energy metabolism [21], however, the precise function in skeletal muscle has yet not been elucidated. FKBP3 was profoundly downregulated. FKBP3 is involved in protein folding and has been linked to myoblast viability and muscle regeneration, and thus has been postulated as a potential regulator of muscle adaptation [22]. In IOPD, the enzyme D‐dopachrome decarboxylase (DDT) and TMEM38A (TRIC), a transmembrane protein essential for calcium handling, necessary for muscle contraction, were highly abundant [23]. ARL8B was highly downregulated in IOPD. ARL8B is a small GTPase involved in lysosomal motility in skeletal muscle. Along with Rab7, ARL8B controls the maturation of the fusion of endosomes and lysosomes [24]. Detailed information of all DAPs is provided in Supporting Information S1: Tables 4 and 5.

### 3.6 The role of protein regulation in relation to disease progression in LOPD

This study is distinguished by its substantial sample size, which comprises a large number of skeletal muscle specimens from individuals with Pompe disease based on different genotypes. The majority of muscle samples included were characterised by a pathology score, which provided an opportunity to analyse protein regulation in relation to the disease progression [4, 5]. The Pompe score showed a significant higher score in IOPD (mean 2.7; 1.7–4) compared to LOPD (mean 0.72; 0.4–2.23); *p* = 0.000004 (Figure 4A, Table 1) highlighting the stronger alterations in skeletal muscle in IOPD. We performed a linear regression analysis on 20 LOPD samples at different Pompe score. Eighty‐four proteins showed an initial *p*‐value of less than 0.05. Correction of *p*‐values refers multiple testing. As a total of 823 proteins were included in the regression model. The initial *p*‐values were adjusted using FDR as correction for multiple testing. Four proteins revealed a significant positive correlation with the Pompe score: vesicle‐associated membrane protein B (VAPB; *p* = 0.024; *r* = 0.79), ribosomal protein S9 (RPS9; *p* = 0.044; *r* = 0.74), beta‐taxilin (TXLNB; *p* = 0.044; *r* = 0.74) and dehydrogenase/reductase SDR family member 7B (DHRS7B; *p* = 0.044; 0.74) (Figure 4B).

**FIGURE 4:**
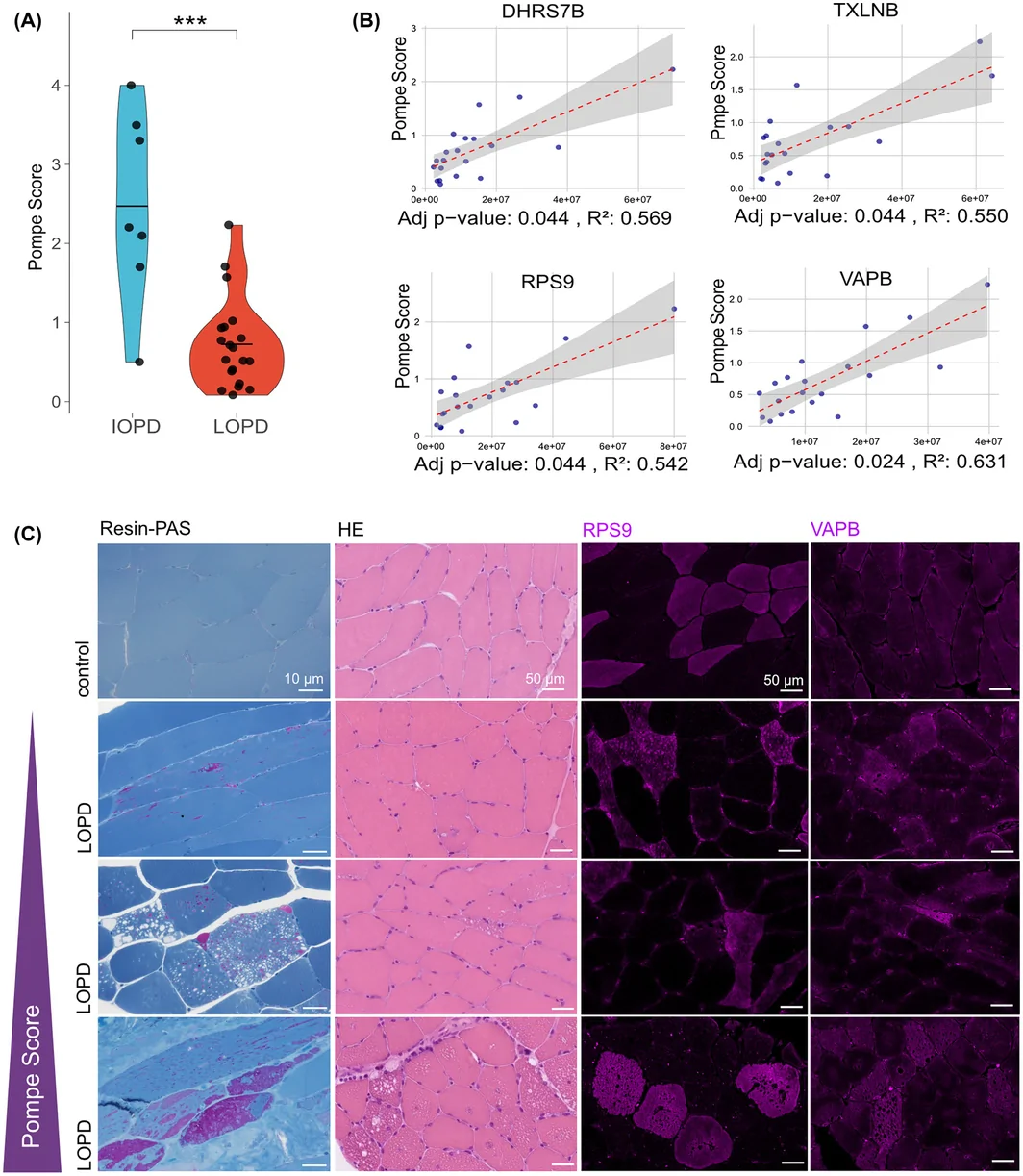
Correlation of protein expression with disease progression in LOPD samples. The Pompe score indicating muscle pathology was significantly higher in IOPD (mean 2.7; 1.7–4) compared to LOPD (mean 0.72; 0.4–2.23); *p* = 0.000004 (A). Regression analysis of 20 LOPD samples with different pathology scores revealed a significant positive correlation of four proteins: DHRS7B, TXLNB, RPS9 and VAPB (B). Representative Resin‐PAS and H&E stained sections revealed disease progression in LOPD samples with mild, moderate and high Pompe score. Immunofluorescence staining confirmed a stronger expression of RPS9 and VAPB in LOPD muscle fibres with higher disease progression (C). ***p < 0.0001.

Based on these findings, we performed immunofluorescence studies with antibodies against VAPB and RPS9 on three representative LOPD muscle biopsy specimens with different stages of disease progression (Pompe score) and one control. Resin‐PAS and H&E sections illustrated the muscle pathology, showing a higher number of glycogen deposits and vacuoles in biopsies with a higher Pompe score. Immunostaining of RPS9 in healthy muscle fibre revealed nuclear staining and stronger cytoplasmic expression in fast fibre with co‐expression of myosin heavy chain (MHC) 2a isoform consistent with a higher turnover of ribosomal proteins (Supporting Information S1: Figure 2) [25]. Hereby, the abundance of RPS9 positive aggregates was increased in LOPD samples with a higher degree of pathology (Figure 4C). Of note, RPS9 positive aggregates were present in both slow and fast vacuolated LOPD fibres (Supporting Information S1: Figure 2). Immunofluorescence staining with an antibody targeting VAPB showed weak ubiquitous cytoplasmic and nuclear expression in healthy muscle fibres. However, in the three LOPD muscle biopsies, VAPB was abundant in small vacuoles in the vacuolated muscle fibres (Figure 4C, Supporting Information S1: Figure 2). The regression analysis on five IOPD samples with different Pompe scores did not reveal any significant results, likely because of a lower number of samples analysed and lower variability in the degree of pathology.

### 3.7 Downregulation of sarcomeric proteins in LOPD

The sarcomere is the smallest contractile unit of striated muscle cells and is responsible for muscle contraction. It is composed of filament systems, including thin (actin) and thick (myosin) filaments [26]. In skeletal muscle 50% of the proteins belong to sarcomeric structures [27, 28]. A previous study described that structural and sarcomeric proteins are deregulated in ERT‐naive LOPD samples [14]. In our study of LOPD samples, 56% of downregulated DAPs belonged to sarcomere and structural proteins involving proteins related to thick and thin filamins (Figure 5A). Skeletal muscle fibres are classified as slow twitching (type 1) or fast twitching (type 2) fibres, delineated by the expression of specific MHC isoforms [25]. DAPs associated with fast fibres (MYH1, MYH2 and MYH4) were more affected than DAPs associated with slow fibres. Comparison of selected sarcomere‐associated proteins such as MYBPC1 (slow type), MYBPC2 (fast type), TPM1, TPM2, TPM3, NEB, MYL5 and MYL6B showed significantly lower abundance in LOPD samples compared to controls. The damage marker XIRP2 was upregulated (Supporting Information S1: Table 5; Figures 2C and 5A). The increased expression of XIRP2 in LOPD was confirmed by immunofluorescence staining (Figure 5B). The electron microscopy findings are consistent with a disturbance of the sarcomeric structures. Different stages of disturbance to the myofibrillar architecture, ranging from mild Z‐band alterations to severe destruction of sarcomeric structures are shown in LOPD muscle fibres (Figure 5C).

**FIGURE 5:**
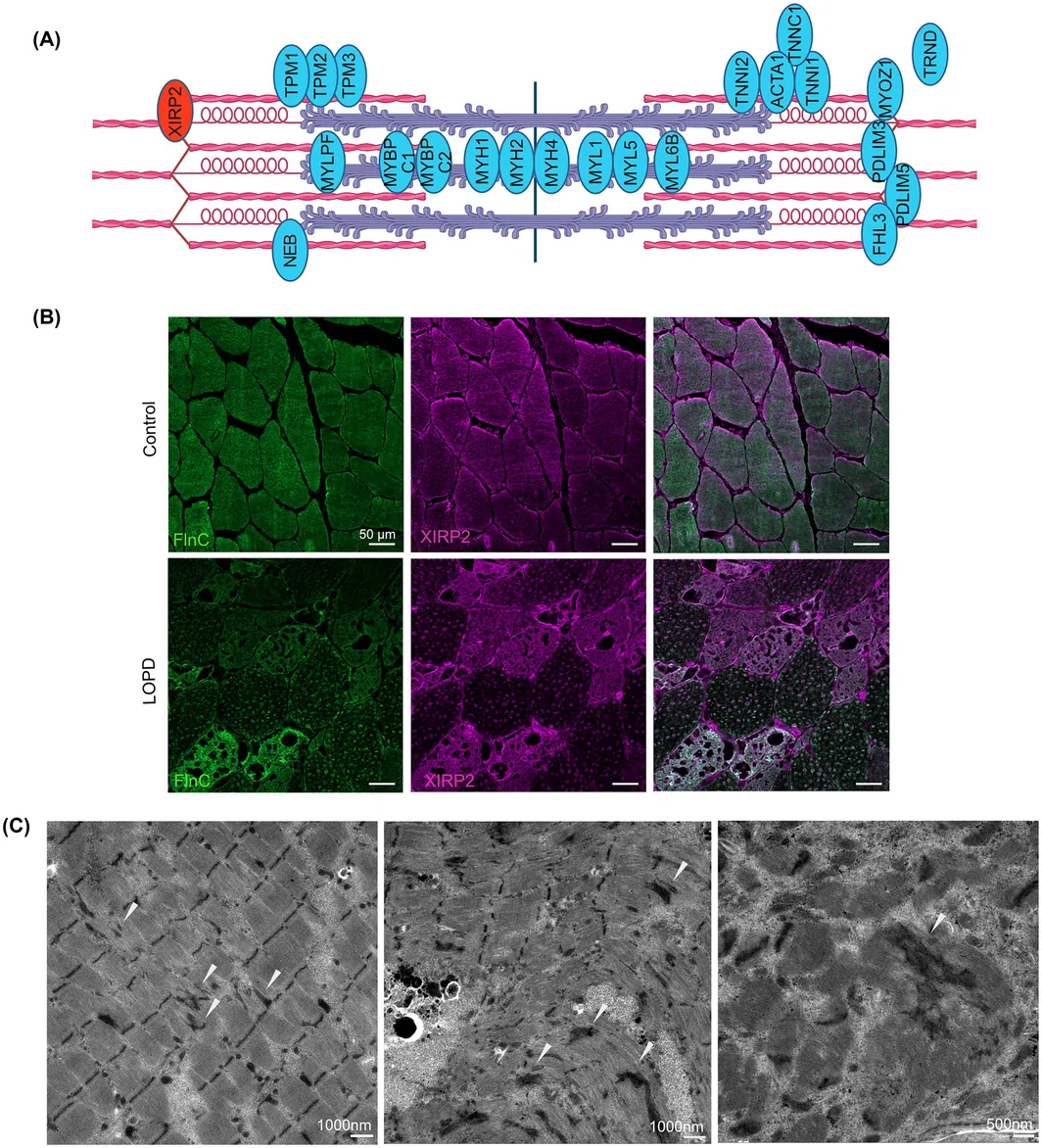
Contractile function in LOPD. Schematic sarcomeric structure depicting the localisation of significantly downregulated (blue) and upregulated proteins (red) indicates involvement of Z‐band, and thick and thin filament proteins (A). Immunofluorescence with antibody against muscle damage marker XIRP2 showed increased expression in vacuolated LOPD muscle fibres (B). Electron microscopy revealed sarcomeric pathology from Z‐band streaming up to severe Z‐band alterations in the muscle fibre (arrowheads) (C).

### 3.8 Deregulation of molecular function associated with muscle contraction and development in IOPD

In IOPD, gene set enrichment analysis revealed downregulation of genes associated with muscle cell development and differentiation, muscle system processes, muscle contraction and adaptation, and cell–cell signalling by Wnt and canonical Wnt signalling pathways (Supporting Information S1: Figure 3a). Electron microscopy showed muscle fibres with severe loss of sarcomeres and mitochondrial aggregates revealed that muscle fibres had undergone a loss of myofibrils. In other muscle fibres, intermyofibrillar mitochondria were increased in number with increased variation of size and shape (Supporting Information S1: Figure 3c).

DAPs associated with contractile function and the GO term ‘muscle system process’ were upregulated in IOPD compared to controls. These included alpha cardiac actin (ACTC1), calmodulin (CALM2), trimeric intracellular cation channel type A (TMEM38), LIM and cysteine‐rich domains protein 1 (LMCD1), triadin (TRDN) and small muscle protein (SMPX) (Supporting Information S1: Figure 2c and Supporting Information S2: Table 3). The cardiac isoform of alpha‐actin ACTC1 is expressed in regenerating skeletal muscle [29, 30]. LMCD1 is a transcription cofactor that regulates calcium and muscle hypertrophy and is a positive regulator for muscle mass [31]. SMPX regulates muscle growth and repair [32, 33]. TRDN and TMEM38A are both involved in calcium release, which is crucial for skeletal muscle contractility [34].

### 3.9 Altered autophagic regulation in LOPD and IOPD

In line with the pathophysiological accumulation of glycogen within lysosomes, impaired autophagy occurs in both IOPD and LOPD, and the accumulation of autophagosomes may impair myofibre function, implicating autophagy as a contributor to the disease mechanism [35]. However, the exact molecular basis of the impaired autophagy‐lysosomal system is still not fully understood in Pompe disease. RNA‐Seq analysis revealed that the hallmark term associated with MTORC1 signalling was upregulated in LOPD compared to controls (Figure 6A). Proteomic analysis showed that starch‐binding domain‐containing protein 1 (STBD1) was significantly downregulated in LOPD. STBD1 plays a role in the glycogen‐selective autophagy process, ‘glycophagy’ by glycogen binding and its intracellular trafficking to lysosomes [36]. In Pompe skeletal muscle, lysosomal activity seems to be increased as molecularly indicated by strong expression of acid phosphatase and Lyosome‐associated membrane protein 2 (LAMP2) [4, 5]. In the proteomic analysis, LAMP2 showed a tendency for upregulation in LOPD, but did not reach statistical significance (Supporting Information S1: Table 5; Figure 6B). However, immunofluorescence staining of LAMP2 revealed stronger expression in muscle fibres with more severe pathology revealing increased lysosomal activity (Supporting Information S1: Figure 4). Increased LAMP2 expression in Pompe disease likely represents a compensatory response to lysosomal dysfunction and autophagic stress, reflecting expansion of dysfunctional lysosomal compartments rather than enhanced lysosomal activity.

**FIGURE 6:**
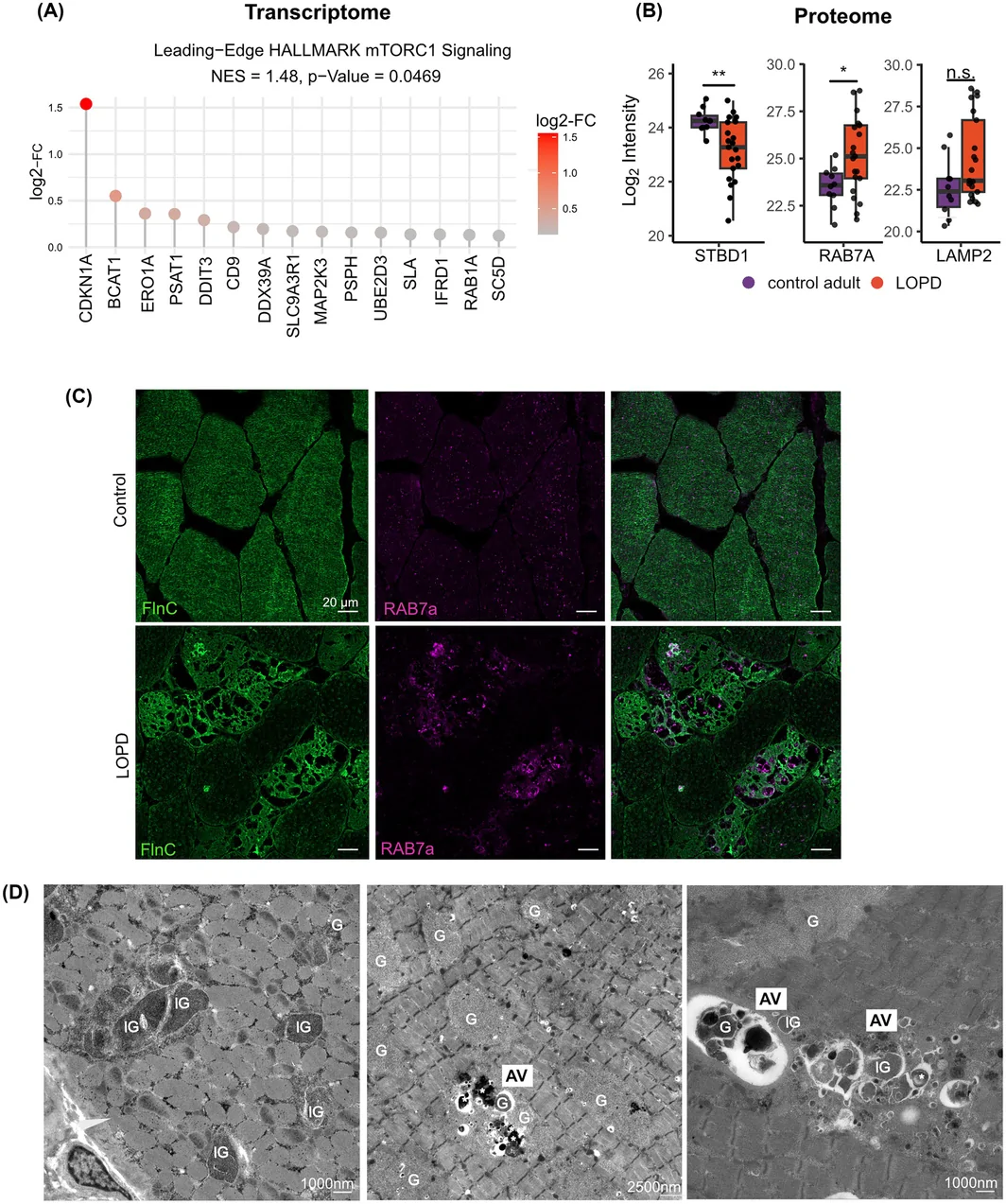
Lysosomal and autophagy processes in LOPD. Pathway analyses with top 15 leading edge genes of significant upregulated hallmark term ‘mTOR signaling’ (A). Box plots indicating significant deregulation of DAPs: Significant lower abundance of STBD1 and significant higher abundance of chaperone‐assisted selective autophagy (CASA) associated protein RAB7A in LOPD compared to controls. The lysosomal marker LAMP2 was higher expressed in LOPD without reaching a significant difference (B). Immunofluorescence studies with antibody against RAB7a showed subtle puncta in healthy muscle and confirmed a strong expression in vacuolated LOPD muscle fibres (C). Electron microscopy showed lysosomal (lG) and extra‐lysosomal glycogen (G) and autophagic vacuoles (AV) with myelin‐like electron dense inclusions (*) in LOPD (D). NES, normalised enrichment score. *p < 0.05; **p < 0.01; ***p < 0.001, n.s. not significant.

In healthy skeletal muscle, removal of tension induced aggregated proteins is crucial for cell maintenance in striated muscle fibres [37, 38]. In a recent study, it has been demonstrated that RAB7A is essential for chaperone‐assisted selective autophagy (CASA) in skeletal muscle [39]. RAB7A was significantly upregulated in our LOPD samples (Figure 6B). Immunostaining using antibodies against RAB7A revealed minimal cytoplasmatic abundance in control specimens, but an increase in vacuolated LOPD muscle fibres (Figure 6C). In a previous study, we have demonstrated increased BCL2‐associated athanogene 3 (BAG3) expression in LOPD samples [5]. BAG3 is a key player of CASA and mediates degradation of unfolded and damaged filamin [37, 38]. Neither on gene nor protein level was BAG3 significantly deregulated in the Pompe samples. However, immunofluorescence revealed BAG3 aggregates in vacuolated muscle fibres of three LOPD samples. Muscle fibres with advanced disease progression—as indicated by increased LAMP2 abundance—and the presence of large vacuoles in H&E‐stained sections contained a larger number of BAG3 aggregates (Supporting Information S1: Figure 4). In line with these molecular observations, electron microscopy of LOPD muscle samples revealed enlarged lysosomes containing glycogen, as well as extra‐lysosomal glycogen deposits and large vacuoles containing myelin‐like material resembling autophagosomes (Figure 6D).

In IOPD, transcriptomics revealed upregulation of heat shock proteins *HSPA6 and 7*, and *LGALS3* (galectin3), when compared to controls (Figure 7A). ARL8B, which is associated with lysosomal transport and vesicle fusion, was downregulated in IOPD (Figure 7B). Immunofluorescence studies confirmed the upregulation of galectin3 in diseased IOPD muscle fibres compared to controls (Figure 7C). Galectin3 abundance was also increased during disease progression in LOPD samples (Supporting Information S1: Figure 5). Electron microscopy identified the presence of large autophagic vacuoles in the majority of IOPD muscle fibres, indicating strongly upregulated autophagic processes (Figure 7D).

**FIGURE 7:**
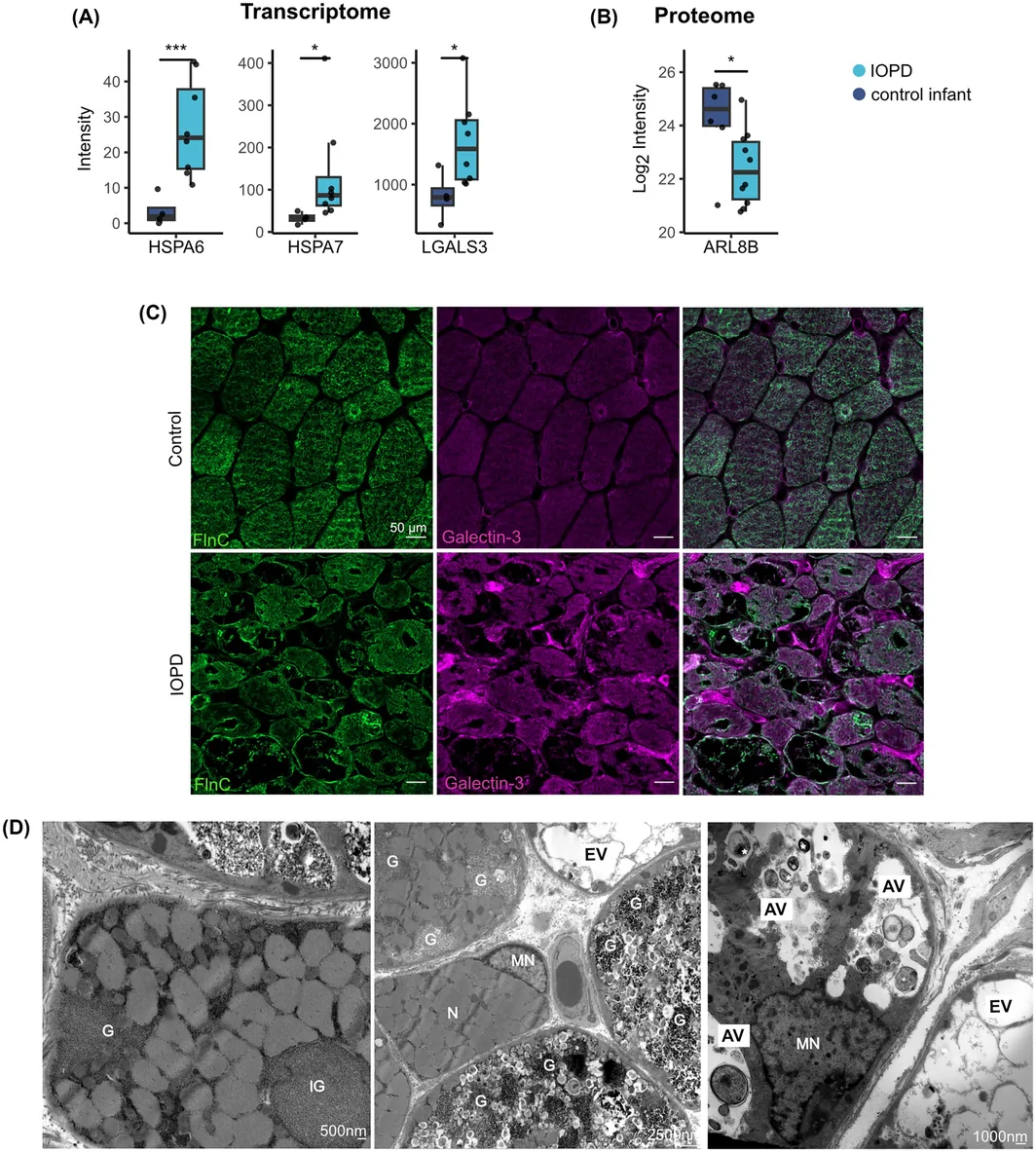
Autophagic and lysosomal regulation in IOPD Box plots indicating significant upregulation of genes encoding heat shock proteins HSPA6 and HSPA7, and galectin3 (LGALS3) in IOPD (A). ARL8B, a protein associated with lysosomal transport, was downregulated in IOPD (B). Immunofluorescence studies with antibody against galectin confirmed higher expression in vacuolated IOPD muscle fibres in comparison to healthy muscle fibres (C). Electron microscopy showed IOPD muscle fibres with strong alterations close to fibres with normal striated appearance (N). The muscle fibres revealed lysosomal (lG) and extra‐lysosomal glycogen (G) accumulation and large autophagosome vacuoles (AV) with electron dense material (*) and empty vacuoles (EV); myonucleus (MN) (D). *p < 0.05; **p < 0.01; ***p < 0.001.

### 3.10 Deregulation of metabolic and mitochondrial processes in LOPD and IOPD

In LOPD and IOPD samples, GO‐term based pathway analysis revealed downregulation of genes associated with metabolic processes with a greater number of processes involved in IOPD. In LOPD, mitochondrial gene expression, energy derivation by oxidation of organic compounds, ncRNA metabolic process and 2‐oxoglutarate metabolic processes were downregulated (Supporting Information S1: Figure 6a). In IOPD, genes associated with energy derivation by oxidation of organic compounds, mitochondrial matrix, cellular glucan metabolic process, proteasome‐mediated ubiquitin‐dependent protein catabolic process, mRNA processing, were significantly lower expressed compared to control (Supporting Information S1: Figure 6b).

The molecular findings were corroborated by the presence of aggregates of polymorphic mitochondria at the ultrastructural level (Supporting Information S1: Figure 6c,d). Proteins associated with energy and metabolism showed differential expression in IOPD and LOPD. Proteins linked to glycolysis were either downregulated (ENO2) or overexpressed (HK1, GBE1) in muscle from LOPD patients (Figure 2A,C, Supporting Information S1: Tables 4 and 5).

### 3.11 Upregulation of immune cell response in LOPD and IOPD at transcriptomic level

Our pathway analysis using GO and Hallmark revealed deregulation of terms associated with immune regulation: In LOPD samples, genes associated with the GO terms ‘adaptive immune response’ (adaptive immune response, mononuclear cell differentiation, T‐cell activation, activation of immune response, B‐cell activation, regulation of lymphocytic activation, positive regulation of immune response, humoral immune response, B‐cell differentiation), ‘regulation of execution phase of apoptosis’ and ‘B cell proliferation’ were upregulated (Supporting Information S1: Figure 7a). The Hallmark terms ‘inflammatory response,’ ‘TNF alpha signaling via NFKB,’ ‘complement’ and ‘interferon gamma response’ were upregulated (Supporting Information S1: Figure 7b). Immunohistochemistry with antibodies against MHC class I revealed a stronger abundance of MHC class I on muscle fibres in a biopsy with more severe Pompe pathology consistent with an activated immune response [40]. In addition, few endomysial T‐lymphocytes can be detected (Supporting Information S1: Figure 7c).

In IOPD samples, GO terms ‘immune response‐regulating signaling pathway’ (immune response‐regulating signalling pathway, positive regulation of immune response, adaptive immune response, activation of immune response, immune response‐regulating cell surface receptor signalling pathway, antigen receptor mediated signalling pathway) (Supporting Information S1: Figure 7d), and the Hallmark term ‘interferon alpha and gamma response’ were upregulated (Supporting Information S1: Figure 7e).

On proteomic level, only in LOPD, peptidyl‐prolyl cis‐trans isomerase (FKBP3), a protein associated with immune cell processes was downregulated (Figure 3A,C, Supporting Information S1: Table 5).

Figure 8 provides a summary of the regulatory muscle processes in IOPD and LOPD at the transcriptomic and proteomic levels.

**FIGURE 8:**
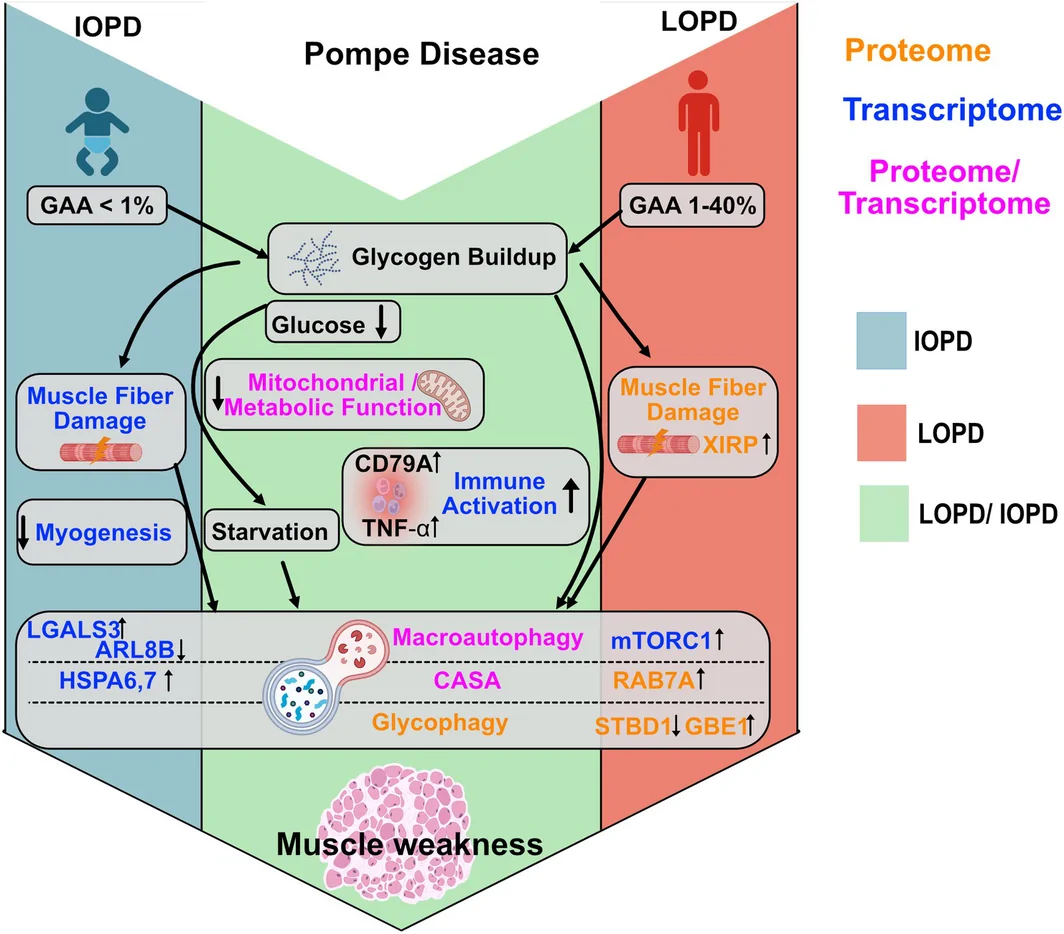
Schematic overview of deregulated muscle processes in LOPD and IOPD on transcriptomic and proteomic level.

### 3.12 Strong regulatory effects of ERT treatment in IOPD on transcriptomic and proteomic level

To explore the effect of ERT treatment on gene expression in IOPD, we calculated RNA expression based on a previously described method that described the effect of ERT on LOPD samples [13]. Using this approach, we observed up‐ and downregulation of DEGs in ERT naive IOPD (baseline) and in treated IOPD compared to controls. ERT treatment reduced the expression of genes that were upregulated in ERT‐naive IOPD and increased the expression of genes that were downregulated in ERT‐naive IOPD. However, expression levels did not fully return to baseline (Figure 9A).

**FIGURE 9:**
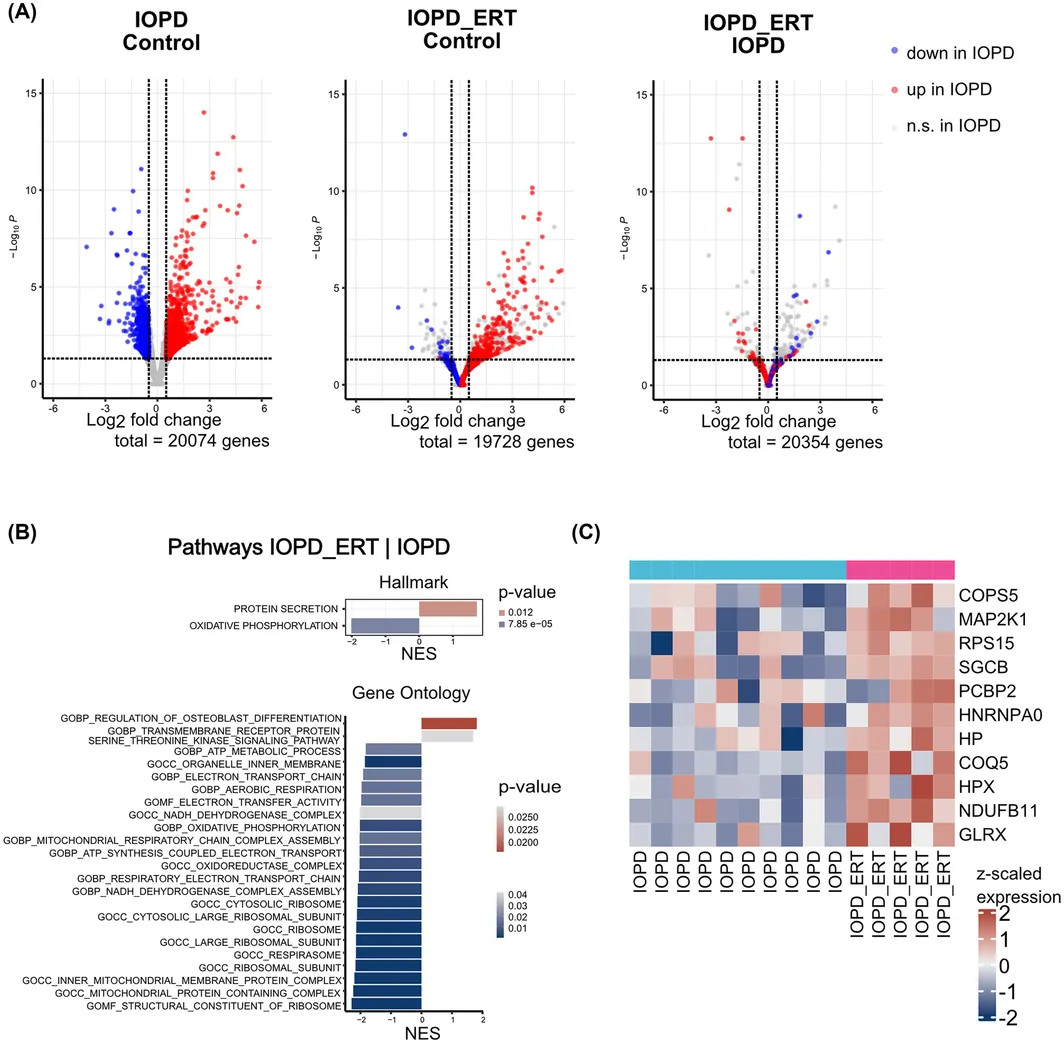
Effect of ERT in IOPD on transcriptomic and proteomic level. Positive effect of ERT on gene expression in IOPD demonstrated with volcano plot. Genes upregulated in naive IOPD (red) were downregulated in IOPD with ERT compared to naive IOPD (red) (A). Pathway analysis (Hallmark) showed an upregulation of genes associated with pathways ‘protein secretion’ and downregulation of ‘oxidative phosphorylation’ in IOPD with ERT. GO analysis revealed downregulation of genes associated with ‘mitochondrial’ and ‘ribosomal’ terms in IOPD with ERT (B). Heatmap of representative DAPs regulated in IOPD with ERT treatment (C).

Additional Hallmark pathway analysis revealed that the ‘protein secretion’ pathway was upregulated and the ‘oxidative phosphorylation’ pathway was downregulated under ERT treatment. GO analysis further supported these findings by showing downregulation of pathways related to mitochondrial and ribosomal function under ERT, indicating regulatory effects on metabolic activity (Figure 9B).

So far, the effect of ERT on the proteomic level has been investigated in LOPD but not in IOPD [14]. Comparing ERT‐treated IOPD with IOPD ERT‐naive samples revealed 14 DAPs of which three were down‐ and 11 were upregulated. In line with our RNA‐Seq data, DAPs upregulated under ERT were associated with developmental (COPS5, MAP2K1 and RPS15), sarcolemmal (SGCB), the immune system (PCBP2, HNRNPA0 and HP) and metabolic/mitochondrial function (COQ5, HPX, NDUFB11 and GLRX1). Downregulated DAPs included proteins with mitochondrial (MT‐ND1 and NDFUB3) and developmental function (DCTN2) (Figure 9C, Supporting Information S1: l. Tables 4, 6 and 7).

## 4 DISCUSSION

This is the first study focusing on both IOPD and LOPD muscle samples by applying a multi‐omics study, muscle morphology and immunofluorescent staining in one approach. Muscle biopsies were analysed retrospectively and comprised a large number of LOPD samples with different degrees of pathology, opening the possibility to decode molecular processes associated with disease progression.

### 4.1 The molecular signature reflects differences in the clinical and morphological phenotype of IOPD and LOPD

Pompe disease is a glycogen storage disease presenting as a severe infantile onset form with a low GAA residual activity and a late onset with higher varying GAA levels [1, 2, 3]. Accordingly, to the differences in the clinical phenotype, the skeletal muscle pathology is more severe in IOPD [4, 5]. The muscle biopsies included in the study showed vacuolated muscle fibres with lysosomal and extra‐lysosomal glycogen accumulation, as well as loss of sarcomeric structures in both IOPD and LOPD, presenting a greater variability in LOPD and stronger pathology in IOPD as confirmed by a Pompe morphology score [4, 5]. Differences were also present at molecular pathways. Consistent with the pathomorphological and clinical presentation, these data indicated stronger differences using transcriptomic and proteomic analysis in IOPD compared to controls than in LOPD. Additionally, LOPD samples showed some overlap with control samples, which is consistent with the finding that some muscle biopsies present with lower pathology.

The transcriptome refers to the complete set of all RNA molecules, both coding and non‐coding, whereas the proteome refers to the complete set of proteins present in cells. Therefore, a combined multi‐omics strategy involving external validation using classical molecular biology assays is a powerful approach for investigating disease mechanisms and improving the reliability of results [41]. In this study, we analysed the proteome based on mass spectrometry and the transcriptome based on RNA‐Seq as separate data planes. Integrating proteomics with transcriptomics provided a multi systems‐level view of RNA and protein networks simultaneously, offering a more comprehensive overview in Pompe disease than examining either layer individually [42]. A higher number of deregulated transcripts compared to proteins were detected in both LOPD and IOPD with a higher number of DEG's in IOPD which might reflect the stronger muscle pathology. Interestingly, the identification of regulatory processes either by transcriptomics or proteomics differed in IOPD and LOPD. For example, downregulation of mitochondrial processes, was indicated in both RNASeq and LC–MS/MS data whereas other processes, such as deregulation of immune processes, were only observed with RNASeq. These findings highlight the importance of combining multiple analytical approaches and validating results with independent methods to better understand the regulatory processes of the disease. Of note, other studies have also reported on low correlation between RNA‐seq and proteomic data [43, 44]. In the proteomic analysis, proteins with high abundance in muscle tissue are more likely to be identified, whereas RNASeq provides a broader view of gene regulation. Therefore, processes modulated by low abundant proteins may be underrepresented [18]. Additionally, the mRNA abundance accounts for only a small percentage of the variation observed at the protein level. This discrepancy may be attributed to the presence of post‐transcriptional control mechanisms, differential protein stability and measurement noise [45, 46]. However, we additional performed a cross‐platform comparison focusing on downstream pathway enrichment which enhances biological interpretability of the results [47]. Our analysis revealed significant deregulation of muscle processes in Pompe disease, involving sarcomeric contractility, cell homeostasis, metabolic activity and immune regulation.

### 4.2 Regulation of muscle fibre contractility and muscle fibre development

Sarcomeric and cytoskeletal proteins present the major proportion in the skeletal muscle proteome [27]. In our study, in both Pompe subtypes processes associated with muscle contractility were deregulated on a molecular level and morphological analysis confirmed structural pathology of the sarcomeres. However, the regulation on gene and protein level was different in IOPD and LOPD: In LOPD, proteomic analysis revealed that the majority of the downregulated DAPs were structural and contractile proteins, including thin and thick filament proteins, which leads to muscle weakness. Hereby, the muscle damage marker XIRP2 was upregulated [48, 49]. Consistent with these findings, ultrastructural morphology showed a variation in myofibrillar disintegration, ranging from mild sarcomeric alterations and Z‐band streaming to severe disruption of the myofibrillar architecture. In previous studies, molecular processes associated with muscle contraction and strong impairment of contractility have been also shown to be deregulated in LOPD [14, 50, 51]. Interestingly, sarcomeric proteins were not fully normalised under ERT, indicating a reduced capability for muscle regeneration under treatment [14]. In our data, a stronger downregulation of fast fibre MHCs indicated that type 2 (glycolytic) muscle fibres were preferably involved. These findings are consistent with other studies showing higher autophagic build‐up in type 2 fibres in LOPD [52]. In comparison to LOPD, such deregulations of sarcomeric proteins were not detected in the IOPD samples. In IOPD skeletal muscles, the myofibrillar architecture is also highly disturbed with loss of sarcomeric structures [4, 8]. Muscle morphology confirmed that myofibrillar architecture was severely affected, suggesting that sarcomeric proteins were also altered in IOPD samples.

One might speculate that the discrepancy between LOPD and IOPD is based on stronger pathology in the IOPD muscle, which reveals a severe loss of sarcomeric proteins and therefore limits the detection with the applied MS methods. However, transcripts associated with sarcomeric function were deregulated in IOPD. *PDLIM*, which may function as an adapter to attract other LIM‐interacting proteins to the cytoskeleton, was downregulated [53]. *TNNT2*, which is supposed to be a cardiac‐specific troponin T isoform, was highly upregulated. TNNT2 expression is significantly increased in skeletal muscles from individuals with skeletal muscle disorders. The expression is closely linked to the degree of disease activity, and the underlying pathophysiology appears to be associated with chronic repair mechanisms [54, 55]. Although our molecular finding supports the concept of TNNT2 as a modulator of activity of muscular diseases, further studies are required to enhance our understanding of TNNT2's function in myopathies and, in particular, its implications in Pompe disease. *COL19A1* was highly upregulated. The transcription of *COL19A1* gradually decreases in differentiating skeletal muscle progenitors, and concomitantly increases myogenin expression consistent with the immature behaviour of skeletal muscle cells in younger subjects [56]. Using pathway analyses, processes of muscle development and differentiation, including Wnt‐signal dependent processes, which are more prominent in healthy infant muscle compared to aged muscle, were downregulated in IOPD indicating a significant loss of muscle fibre function [16]. DAPs associated with ‘contractile function’ and the GO term ‘muscle system process’ were upregulated in IOPD muscles compared to controls. Of note, these DAPs are mainly involved in muscle regeneration, regulation of the calcium balance and contractility and may therefore indicate a higher fibre regenerative capacity in IOPD skeletal muscle.

### 4.3 Protein regulation is linked to disease progression in LOPD

The correlation analysis of LOPD samples presenting with a variable Pompe pathology score identified four significantly regulated proteins indicating processes which are dependent on disease progression. VAPB, RPS9 and TXLNB are involved in biological processes such as vesicular trafficking and protein biosynthesis in skeletal muscle and correlated in our study with a higher pathology score indicating disease progression [57, 58, 59, 60]. The altered abundance of VAPB and RPS9 was validated by immunofluorescent staining, showing a higher protein expression in LOPD samples linked to more severe myopathology. Our data are consistent with other studies, showing increased expression of VAPB together with other autophagy markers in atrophic fibres and sites of focal myofibrillar disintegration in denervated skeletal muscle fibres [60]. DHRS7B, an oxidoreductase located in the ER and related to energy metabolism, has not been described in human skeletal muscle fibres so far [61]. These identified proteins reflect the Pompe pathology, including vesicle transport, protein biosynthesis and cell homeostasis and may therefore serve as tissue‐based biomarkers of disease progression.

### 4.4 Deregulation of autophagy‐lysosomal processes in infantile and adult Pompe disease

Autophagy‐lysosomal function is required for basal myofibre homeostasis [62]. In Pompe pathogenesis, disturbed autophagy processes may accelerate the pathology process [13, 14, 63, 64]. In the Pompe Gaa−/− mouse model, which represents a mixture of LOPD and IOPD features, build‐up of autophagic material correlates with microstructural disturbance of muscle at diffusion MRI [65, 66, 67]. However, it is not fully solved if in Pompe disease autophagy is increased because of accumulation of glycogen and protein debris (increased on‐rate), disturbed autophagosome fusion (reduced off‐rate) or energy shortage, which forces the muscle cells into a starvation state [50, 68].

Our combined data confirm a deregulation of various autophagy‐related processes in Pompe muscle fibres associated with CASA, mTOR processes, lysosomal function and glucophagy. These findings highlight the complexity of the autophagic processes involved in Pompe muscle fibres and indicate that therapeutic options that affect one autophagy cascade may not necessarily improve the pathology.

In healthy skeletal muscle, removal of tension induced aggregated proteins by CASA is crucial for cell maintenance [37, 38]. In our study, we observed a strong deregulation of sarcomeric function in particular in LOPD samples. We propose that CASA might be strongly involved in Pompe disease because of the severe sarcomeric damage and the need for recycling the damaged sarcomeric proteins as it has been shown in other myopathies with strong structural disruption of the myofibrils [69, 70]. With our data we confirmed a deregulation of CASA associated process in LOPD mainly on proteomic level and IOPD on transcriptomic level. BAG3 is a key player in CASA [71, 72, 73]. Mutations in the *BAG3* gene result in a disturbed autophagic regulation in myocytes leading to myopathy of the heart and skeletal muscle [74, 75]. Our immunofluorescence studies and previous data clearly indicated that BAG3 expression on a cellular level rises in proportion to the presence of autophagic vacuoles and the progression of Pompe pathology in LOPD [5]. However, BAG3 was not significantly deregulated at gene or protein level. The formation of BAG3 aggregates in Pompe muscle fibres may thus not be a consequence of increased protein expression. In this case, LC–MS/MS methods that separate analysis of soluble and insoluble fractions separately might be preferable, especially for the detection of insoluble protein aggregates [76]. In comparison, RAB7a was abundant in LOPD samples. RAB7A is involved in the maturation of autophagosomes and participates in their fusion with lysosomes and is essential for CASA in skeletal muscle [39, 77, 78]. Our findings support that RAB7a might serve a role in autophagy processes in diseased human skeletal muscle.

In IOPD samples, HSP's were upregulated on the transcriptomic level. Chaperones of the HSPA family are part of the CASA complex and play an important role in protein quality control systems and targeting of misfolded proteins for degradation [79]. These data confirm the role of CASA processes also in IOPD pathology. At the other hand, ARL8B, a small GTPase involved in lysosomal motility in skeletal muscle which like Rab7 controls maturation function of fusion of endosomes and lysosomes, was downregulated in IOPD compared to controls [24]. A reduction in ARL8B may be linked to lysosomal dysfunction and impaired autophagy. This undermines the role of lysosomal processes in Pompe disease, though further functional research is needed.

Rapamycin complex 1 (mTORC1) plays a major role in autophagy induction in skeletal muscle, and its deregulation leads to pathological alterations of muscle homeostasis and muscle diseases [80]. In pluripotent stem cells (iPSCs) from Pompe patients, mTORC1 activity is impaired, and mTORC1 suppression was proposed as adjunct therapy in Pompe disease [81, 82]. Our study showed that MTORC1 signalling was upregulated at the transcriptomic level in LOPD samples, indicating altered regulation consistent with recent findings [50, 51]. Nutrient deprivation (e.g., low glucose level) inhibits mTORC1 by activation of AMPK, which initiates autophagy via phosphorylation of ULK1 [83, 84]. Thus, we hypothesise that lower residual activity of GAA results in an increased role for autophagy through starvation in IOPD relative to LOPD. However, this hypothesis cannot be substantiated by the data available and requires more pre‐clinical research.

STBD1 is most prevalent in liver and skeletal muscle, which both contain large deposits of glycogen and play a role in the glycogen‐selective autophagy process (glycophagy), by binding glycogen and its intracellular trafficking to lysosomes [36, 85, 86]. The role of STBD1 has not been investigated in human skeletal muscle before. Interestingly, we found a significant downregulation of STBD1 in LOPD samples, indicating that a disturbed glycophagy may aggravate muscle pathology or might be reduced becuase of disturbed glycogen metabolism. In contrast to our data, in Gaa−/−‐Pompe mice, STBD1 expression was increased in skeletal and cardiac muscle [87]. However, our study included muscle samples from adults with severe pathology, whereas Pompe mice mainly show a less severe pathology, which may affect these regulatory processes.

Galectin3, a marker of endo‐lysosomal damage, is upregulated in a Pompe mouse model and may serve as a prognostic autophagy‐related biomarker [88]. In line with this data, our IOPD samples revealed upregulation of *LGALS3* (Galectin3), declaring this protein to be a suitable tissue‐biomarker across species.

### 4.5 Increased immune processes in IOPD and LOPD

Our omics data revealed an increase of gene sets associated with immune processes in both subtypes, confirming previous findings [13, 14, 15, 50, 89]. Notably, *CD79a* was found to be highly expressed in LOPD, which has been only demonstrated in IOPD muscle so far [15]. The upregulation of the proinflammatory cytokine TNF‐α in LOPD may contribute to the loss of protein in the muscle cell and myofibre atrophy [90]. In accordance with the data, we observed an elevated expression of MHCI in LOPD with higher upregulation on muscle fibres exhibiting more severe pathology. In IOPD, gene sets related to interferon‐α signalling—known to play a central role in inflammatory myopathies and to inhibit myoblast differentiation [91]—were upregulated. Dysregulation of autoimmune processes may therefore exacerbate muscle fibre damage and impair muscle regeneration in Pompe disease.

### 4.6 Deregulation of metabolic processes in IOPD and LOPD

In our IOPD and LOPD samples, GO analyses revealed downregulation of pathways associated with metabolic processes consistent with previous studies on LOPD samples [13, 50, 51, 92]. Our data revealed a significant downregulation of OXPHOS and other mitochondrial pathways, confirming a severe impairment of cellular respiration and energy metabolism in Pompe disease [13]. In accordance with this molecular finding, ultrastructural analysis confirmed mitochondrial pathology.

While increased lipid and protein metabolism has been proposed as a compensatory response to impaired glucose utilisation in LOPD [13], our findings indicate downregulation of adipogenic and fatty acid metabolic pathways in both LOPD and IOPD. This may reflect a transition from early compensatory metabolic remodeling to a state of metabolic insufficiency in muscles with more severe pathology. Proteins associated with glycolysis were found to be down‐ and upregulated in LOPD. This is in line with a previous study that described up‐ and downregulation of proteins involved in glycolysis and gluconeogenesis in LOPD [14]. GBE1 was upregulated in our LOPD samples. As demonstrated in Gaa−/− Pompe mice, GBE1 controls glycogen biosynthesis, indicating a disturbance in cytoplasmic glycogen metabolism [93]. Extra‐lysosomal glycogen was identified in our resin sections and at the ultrastructural level. This accumulation may contribute to the muscle pathology by disrupting myofibrillar architecture [7].

### 4.7 Age‐dependent regulation of muscle processes in infantile and adult Pompe disease

In older subjects, skeletal muscle homeostasis is altered, including deregulation of sarcomeric, metabolic and autophagic function [16, 94, 95]. Our previous study analysing skeletal muscle biopsies from healthy infants compared to adults, revealed a decrease in sarcomeric, developmental, immune and cell maintenance processes in adults [16]. Our LOPD cohort includes adult patients aged 26–78 years. Therefore, physiological aging may aggravate muscle pathology in adult Pompe patients for instance because of decreased sarcomeric function. Myogenesis and muscle development were downregulated in IOPD samples, which included very young patients (0.1–0.8 years) and thus contained immature muscle fibres, whereas no such deregulation was observed in LOPD. This suggests an age‐dependent impairment of muscle development in IOPD, consistent with recent findings by Severa and colleagues, who reported higher regenerative capacity in younger compared to older LOPD patients [96].

### 4.8 Normalisation on gene regulation with ERT in IOPD

The effect of ERT on the molecular level has not yet been investigated in detail in IOPD muscle samples. A previous study analysing the effect of ERT in IOPD using microarrays showed regulation of pathways related to muscle regeneration, inflammation and fibrosis [15]. However, controls used were much older than the IOPD patients, and microarrays have lower resolution compared to RNA‐Seq. In our study, we observed in muscle samples from IOPD patients treated with ERT regulatory effects similar to those reported before on LOPD samples [13]. Our data show that ERT has a positive effect on the function of skeletal muscles in IOPD, including the upregulation of developmental, sarcomeric, immune system and metabolic processes.

## 5 STRENGTHS AND LIMITATIONS

The skeletal muscle samples included in our study were collected retrospectively. This enabled us to conduct a multi‐omics analysis of a large cohort of IOPD and LOPD samples without ERT, as well as LOPD samples with varying pathological severity. We also included samples of IOPD patients treated with ERT. These samples were collected as part of therapeutic interventions, so the time period between the first and second biopsy was not standardised. Because of the limitations of sample size, additional validation with western blot analysis could not be performed.

## 6 CONCLUSIONS

In our study, we have demonstrated that a comprehensive analysis, incorporating a multi‐omic approach in addition to immune fluorescence and morphological techniques, increases understanding of the underlying pathophysiology in Pompe disease. The study highlights differences in the regulation of skeletal muscle processes at the transcriptomic and proteomic level in infantile and adult Pompe disease.

## AUTHOR CONTRIBUTIONS

**ASchän**, **ARoo** and **ASchai** designed the study, analysed data and drafted the manuscript. **ASchai**, **ASchän**, **MB**, **ARoo**, **AHe** and **TP‐K** performed data analysis. **JH** and **DOF** discussed data. **PFMvdV**, **ARot** and **KL** performed immunofluorescence studies. **AHa**, **AM**, **ARos**, **CK**, **WS** and **HHK** provided clinical data. All authors have read and approved the final manuscript.

## FUNDING INFORMATION

For this study unrestricted research funding was given to ASchän by Sanofi (SGZ‐2022‐13788). This study was supported by the ‘Ministerium für Kultur und Wissenschaft des Landes Nordrhein–Westfalen,’ ‘Regierenden Bürgermeister von Berlin—Senatskanzlei Wissenschaft und Forschung,’ and ‘Bundesministerium für Forschung, Technologie und Raumfahrt.’ The European Regional Development Fund (ERDF) financed parts of this study in the framework of the NME‐GPS project (NME‐GPS; grant to ARoo).

## CONFLICT OF INTEREST STATEMENT

The authors declare that they have no competing interests.

## ETHICS STATEMENT

Written informed consent was obtained from the subjects or subject's parents. This project was approved by the local ethic committee (AZ 07/09 Justus Liebig University Giessen) and conducted in accordance with the Declaration of Helsinki.

## Supporting information


**Data S1.** Supporting Information.

**Data S2** Supporting Information.

## Data Availability

Access to the transcriptomic and proteomic data will be available upon reasonable request to the corresponding author.
