# Supplementary material for: Identifying regulatory muscle processes in infantile and adult Pompe patients using a multi‐omic and morphological approach
Source: Brain Pathol. 2026 Oct 7:e70155. Online ahead of print. doi: 10.1111/bpa.70155 (PMC13645463; doi:10.1111/bpa.70155)
Supplement: Supplementary file 1 [file BPA-9999-e70155-s002.docx]

# **Supplementary information**

# **Identifying regulatory muscle processes in infantile and adult Pompe patients using a multi-omic and morphological approach**

Alexander Schaiter^1^, Keerthika Lohanadan^2^, Alexander Mensch^3^, Andreas Hentschel^4^, Angela Rosenbohm^5^, Cornelia Kornblum^6^, Angela Roth^1^, Tara Procida-Kowalski^7^, Heidrun H. Krämer ^8,9^, Andreas Hahn^10^, Jörg Höhfeld^2^, Dieter O. Fürst^2^, Werner Stenzel^11^, Peter F.M. van der Ven^2^, Andreas Roos^12,13,14^#, Marek Bartkuhn^7,15^#, Anne Schänzer^1,9^#*

*Correspondence author: Anne Schänzer, [anne.schaenzer@patho.med.uni-giessen.de](mailto:anne.schaenzer@patho.med.uni-giessen.de); phone +49 641 99 41184; fax +49 641 99 41189

\# contributed equally to the study

**Material and Methods**

**Transcriptomics**

Total RNA was isolated with the RNeasy^®^ Mini Kit (Qiagen) following the manufacturer’s instructions including on-column DNA removal by DNase digestion using the RNase-free DNase set (Qiagen). Disruption and homogenization of muscle tissue slices were performed in 600 µl Buffer RLT containing DTT by passing the lysate through an RNase-free needle (20G) and syringe until a homogeneous suspension was obtained. Purified RNA was subjected to quality control by capillary electrophoresis (4200 TapeStation, Agilent).

For genome-wide analysis of gene expression, RNA sequencing libraries from isolated mRNA were generated and sequenced by the Genomics and Bioinformatics platform of the Institute for Lung Health (ILH) at the Justus-Liebig-University (JLU) Giessen (Germany). A total amount of 25 ng of RNA per sample was used to enrich for polyadenylated mRNA followed by cDNA sequencing library preparation utilizing the Illumina^®^Stranded mRNA PrepKit (Illumina) according to the manufacturer’s instructions. After library quality control by capillary electrophoresis (4200 TapeStation, Agilent), cDNA libraries were sequenced on the Illumina NovaSeq 6000 platform generating 50 bp paired-end reads. Generally, we sequenced > 20x10^6^ reads per sample ^1^.

**Proteomics**

Muscle sample lysis involved the addition of 200 µl of lysis buffer containing 50 mM Tris–HCl (pH 7.8), 5% SDS, and cOmplete ULTRA protease inhibitor (Roche). The Bioruptor® (Diagenode) was employed for 10 min (30 s on, 30 s off, 10 cycles) at 4 °C to facilitate lysis. To ensure thorough lysis, an additional step involved sonication using an ultra-sonic probe (30 s, 1 s/1 s, amplitude 40%), followed by centrifugation at 4 °C and 20,000 g for 15 min. Protein concentration was determined using the BCA assay as per the manufacturer's instructions. Disulfide bonds were reduced by adding 10 mM TCEP at 37 °C for 30 min, and free sulfhydryl bonds were alkylated with 15 mM IAA at room temperature in the dark for 30 min. For proteolysis, 100 µg protein from each sample underwent S-Trap protocol (Protifi) digestion with a trypsin to protein ratio of 1:20 for 2 h at 37 °C. Proteolysis was halted by acidifying the sample (pH < 3.0) with formic acid.

Complete digestion of all proteolytic samples was verified after desalting using monolithic column separation (PepSwift monolithic PS-DVB PL-CAP200-PM, Dionex) on an inert Ultimate 3000 HPLC (Dionex, Germering, Germany). This involved direct injection of 1 μg sample and a binary gradient (solvent A: 0.1% TFA, solvent B: 0.08% TFA, 84% ACN) ranging from 5 to 12% B in 5 min and then from 12 to 50% B in 15 min at a flow rate of 2.2 μL/min and at 60 °C. UV traces were acquired at 214 nm ^2^.

***DIA-LC-MS/MS analysis***

All samples were analyzed using an UltiMate 3000 RSLC nano UHPLC coupled to a QExactive HF mass spectrometer, and the total amount of peptide applied was always 1 µg. Samples were loaded onto a 75 µm × 2 cm, 100 Å C18 precolumn at 10 µL/min for 20 min, then separated on a 75 µm × 50 cm, 100 Å C18 main column at 250 nL/min using a 120‑min linear gradient from 3% to 45% solution B (A: 99.9% water, 0.1% formic acid; B: 84% acetonitrile, 15.9% water, 0.1% formic acid). The gradient was applied as follows: 3% B for 20 min, 3-35% for 120 min, followed by 3 wash steps, each reaching 95% buffer B for 3 min. After the last wash step, the instrument was allowed to equilibrate for 20 min. MS data acquisition was performed in DIA (data independent acquisition) mode using an in-house generated spectral library. Each analyzed sample was mixed with an appropriate amount of iRT standard (Biognosys). Full MS scans were acquired from 300-1100 m/z at a resolution of 60,000 (Orbitrap) using the polysiloxane ion at 445.12002 m/z as the lock mass. The automatic gain control (AGC) was set to 3E6 and the maximum injection time was set to 20 milliseconds. The full MS scans were followed by 23 DIA windows, each covering a range of 28 m/z with an overlap of 1 m/z starting at 400 m/z and acquired at a resolution of 30,000 (Orbitrap) with an AGC of 3E6 and an nCE of 27 (CID).

**Bioinformatic analysis**

## To ensure the robustness and reproducibility of RNA-Seq and MS data, we followed the Max Planck Institute of Biochemistry`s recommended statistical practises for data pre-processing. We applied a false discovery rate (FDR) > 0.05 correction to all measured variables during both the differential expression analysis and pathway enrichment analyses.

The raw counts from RNA-Seq were processed using the DESeq2 package to normalize the data and identify differentially expressed genes (DEGs) between the experiment groups ^3^. To address noise and variability in the RNA-Seq data, the adaptive shrinkage method “ashr” was applied to estimate gene expression variance toward a common value, thus improving the accuracy of downstream differential expression analysis. For label-free proteomics (MS), raw data obtained via nano-LC-MS/MS in Data Independent Acquisition (DIA) mode were analyzed using the Maxquant software (v2.0.3.0) with a library-based search ^4, 5^. Standard significance thresholds were applied (FDR = 1%), and the "match between runs" option with label-free quantification (LFQ) was used. After filtering out the proteins identified by site, reverse hits, and potential contaminants. Protein groups detected in at least 50% of samples from one group of contrast were included in the subsequent analysis. We imputed the missing values based on a with a witdth of 0.3 and down-shift of 1.8.

We identified significantly differentially expressed genes (DEGs) and differentially abundant proteins (DAPs) using thresholds of log₂ fold changes (>1 or <-1) and adjusted p-values <0.05. Functional annotations of the proteins from UniProt (<https://www.uniprot.org>), GeneCards (https://www.genecards.org/) and the literature databases were used (<https://pubmed.ncbi.nlm.nih.gov>).

Gene functions were obtained using the corresponding R packages, which linked information from the Gene Ontology (GO) and Hallmark databases. Pathway enrichment analysis was performed using the clusterProfiler R package ^6^. This approach was applied to both transcriptomic and proteomic datasets. Gene Set Enrichment Analysis (GSEA) was applied for the transcriptomic data to identify significantly enriched biological pathways using pre-ranked gene lists. This method identified pathways across all measured transcripts, even when individual transcripts did not reach statistical significance. GO and Hallmark were used as the gene set databases ^7, 8^. GO provided a comprehensive view of biological processes, molecular functions and cellular components across various pathways. In contrast, Hallmark focused on a smaller set of key pathways, reducing complexity and highlighting well-defined biological states or processes ^8^. Combining both databases provided detailed functional insights and targeted key pathways.

Data visualization was carried out using the following R packages: ggplot2 for general plotting, Enhanced Volcano for visualizing DEGs and DAPs, networkD3 for Sankey diagrams, and ComplexHeatmap for hierarchical clustering results ^9-12^.

[1] Schaiter A, Hentschel A, Kleefeld F, Schuld J, Umathum V, Procida-Kowalski T, Nelke C, Roth A, Hahn A, Krämer HH, Ruck T, Horvath R, van der Ven PFM, Bartkuhn M, Roos A, Schänzer A: Molecular composition of skeletal muscle in infants and adults: a comparative proteomic and transcriptomic study. Scientific reports 2024, 14:22965.

[2] Burkhart JM, Schumbrutzki C, Wortelkamp S, Sickmann A, Zahedi RP: Systematic and quantitative comparison of digest efficiency and specificity reveals the impact of trypsin quality on MS-based proteomics. Journal of Proteomics 2012, 75:1454–62.

[3] Love MI, Huber W, Anders S: Moderated estimation of fold change and dispersion for RNA-seq data with DESeq2. Genome Biol 2014, 15:550.

[4] Tyanova S, Temu T, Cox J: The MaxQuant computational platform for mass spectrometry-based shotgun proteomics. Nature protocols 2016, 11:2301–19.

[5] Tyanova S, Temu T, Sinitcyn P, Carlson A, Hein MY, Geiger T, Mann M, Cox J: The Perseus computational platform for comprehensive analysis of (prote) omics data. Nature methods 2016, 13:731–40.

[6] Yu G, Wang L-G, Han Y, He Q-Y: clusterProfiler: an R package for comparing biological themes among gene clusters. Omics: a journal of integrative biology 2012, 16:284–7.

[7] Ashburner M, Ball CA, Blake JA, Botstein D, Butler H, Cherry JM, Davis AP, Dolinski K, Dwight SS, Eppig JT, Harris MA, Hill DP, Issel-Tarver L, Kasarskis A, Lewis S, Matese JC, Richardson JE, Ringwald M, Rubin GM, Sherlock G: Gene ontology: tool for the unification of biology. The Gene Ontology Consortium. Nature genetics 2000, 25:25–9.

[8] Liberzon A, Birger C, Thorvaldsdóttir H, Ghandi M, Mesirov JP, Tamayo P: The Molecular Signatures Database (MSigDB) hallmark gene set collection. Cell Syst 2015, 1:417–25.

[9] Gu Z: Complex heatmap visualization. iMeta 2022, 1:e43.

[10] Gu Z, Eils R, Schlesner M: Complex heatmaps reveal patterns and correlations in multidimensional genomic data. Bioinformatics 2016, 32:2847–9.

[11] Blighe K, Rana S, Lewis M: EnhancedVolcano: Publication-ready volcano plots with enhanced colouring and labeling. <https://githubcom/kevinblighe/Enhanced/Volcano> 2018, 1.

[12] Wickham H, Wickham H: Getting Started with ggplot2. ggplot2: Elegant graphics for data analysis 2016:11–31.

**Supplementary Table 1:** Antibodies

| **Antibody** | **Species** | **Manufacturer / Reference** | **Dilution**  **IF**/DABKryo |
| --- | --- | --- | --- |
| RAB7A | Rabbit | Abcam ab137029 | 1:200 |
| BAG3 | Rabbit | Proteintech 10599-I-AP | 1:100 |
| Desmin | Mouse | DAKO M0760 | 1:200 |
| Desmin | Rabbit | Abcam ab32362 | 1:200 |
| Myotilin | Mouse | Leica Novocastra | 1:50 |
| LAMP2 | Mouse | Abcam ab25631 | 1:100 |
| P62 | Mouse | BD Transd. Lab. 610832 | 1:200 |
| LC3 | Mouse | Nano Tools 0231-100/LC3-5F10 | 1:100 |
| RR90 (Filamin) | Mouse | van der Ven et al., 2000 | 1:30 |
| Galectin-3 | Rabbit | Proteintech 14979-1-AP | 1:100 |
| VAPB | Rabbit | Proteintech 14477-1-AP | 1:300 |
| RPS9 | Rabbit | Proteintech 18215-1-AP | 1:100 |
| BA-F8 (MHC1) | Mouse | DSHB | 1:10 |
| SC71 (MHC2a) | Mouse | DSHB | 1:10 |
| 7700 (XIRP2) | Rabbit | Kley et al., 2013 | 1:50 |
| MHCI (W6/32) | Mouse | Dako / M0736 | 1: 500 (DAB) |
| CD3 | Rabbit | Roche / 790 4341 | Ready to use (DAB) |

P.F.M. van der Ven, W.M.J. Obermann, B. Lemke, M. Gautel, K. Weber, D.O. Fürst, Characterization of muscle filamin isoforms suggests a possible role of γ-filamin/ABP-L in sarcomeric Z-disc formation, Cell Motil. Cytoskeleton 45 (2000) 149–162, https://doi.org/10.1002/(SICI)1097-0169(200002)45:2<149:AID-CM6>3.0.CO; 2-G.

D.O. Fürst, M. Osborn, R. Nave, K. Weber, The organization of titin filaments in the half-sarcomere revealed by monoclonal antibodies in immunoelectron microscopy: a map of ten nonrepetitive epitopes starting at the Z line extends close to the M line, J. Cell Biol. 106 (1988) 1563–1572, <https://doi.org/10.1083/jcb.106.5.1563>.

R.A. Kley, A. Maerkens, Y. Leber, V. Theis, A. Schreiner, P.F.M. van der Ven, J. Uszkoreit, C. Stephan, S. Eulitz, N. Euler, J. Kirschner, K. Müller, H.E Meyer, M. Tegenthoff, D.O. Fürst, M. Vorgerd, T. Müller, K. Marcus. A combined laser microdissection and mass spectrometry approach reveals new disease relevant proteins accumulating in aggregates of filaminopathy patients. Mol Cell Proteomics 12 (2013) 215–227. https://doi.org/10.1074/mcp.M112.023176

**Supplementary Table 2:** Samples

| **Patient** | **Group** | **Proteomic** | **Transcriptomic** | **IF** | **TEM** |
| --- | --- | --- | --- | --- | --- |
| I1 | IOPD | X | X |  |  |
| I2 | IOPD | X | X |  |  |
| I3a | IOPD | X |  |  |  |
| I3b | IOPD_ERT | X | X |  |  |
| I4a | IOPD | X |  |  |  |
| I4b | IOPD_ERT | X |  |  |  |
| I5 | IOPD | X | X |  |  |
| I6 | IOPD | X | X |  |  |
| I7 | IOPD | X | X |  |  |
| I8 | IOPD |  | X |  | X |
| I9 | IOPD | X | X |  |  |
| I10a | IOPD | X | X |  |  |
| I10b | IOPD_ERT | X |  | X |  |
| I10c | IOPD_ERT | X |  |  |  |
| I11 | IOPD | X |  |  |  |
| I12 | IOPD_ERT | X |  |  |  |
| I13 | IOPD_ERT | X |  |  |  |
| L1 | LOPD | X | X |  | X |
| L2 | LOPD | X |  |  |  |
| L3 | LOPD | X |  |  |  |
| L4 | LOPD | X |  |  |  |
| L5 | LOPD | X |  |  |  |
| L6 | LOPD | X |  |  |  |
| L7 | LOPD | X |  |  |  |
| L8 | LOPD | X | X |  |  |
| L9 | LOPD | X |  | X |  |
| L10 | LOPD | X | X |  |  |
| L11 | LOPD | X | X |  |  |
| L12 | LOPD | X | X |  |  |
| L13 | LOPD | X | X |  | X |
| L14 | LOPD | X | X |  |  |
| L15 | LOPD | X | X |  |  |
| L16 | LOPD | X |  | X |  |
| L17 | LOPD | X |  | X |  |
| L18 | LOPD | X |  | X | X |
| L19 | LOPD | X | X |  |  |
| L20 | LOPD | X | X |  |  |
| L21 | LOPD | X | X |  |  |
| L22 | LOPD |  |  | X |  |
| L23 | LOPD |  |  | X |  |
| CI1 | Control_infant | X | X |  |  |
| CI2 | Control_infant | X |  | X |  |
| CI3 | Control_infant | X |  |  |  |
| CI4 | Control_infant | X | X |  |  |
| CI5 | Control_infant | X |  |  |  |
| CI6 | Control_infant | X | X |  |  |
| CI7 | Control_infant |  | X |  |  |
| CA1 | Control_adult | X | X |  |  |
| CA2 | Control_adult | X |  |  |  |
| CA3 | Control_adult | X | X |  |  |
| CA4 | Control_adult | X | X |  |  |
| CA5 | Control_adult | X |  |  |  |
| CA6 | Control_adult | X | X |  |  |
| CA7 | Control_adult | X | X |  |  |
| CA8 | Control_adult | X | X |  |  |
| CA9 | Control_adult | X |  |  |  |
| CA10 | Control_adult | X | X |  |  |
| CA IF | Control adult |  |  | X |  |

**RESULTS**

**Supplementary Table 3: RNASeq Data – Excel table**

**Supplementary Table 4:** DAPs in IOPD compared to age matched controls

| **Protein** | **Name** | **Function**  **(selected)** | **log2 Fold-change** | **Adjusted p-Value** |
| --- | --- | --- | --- | --- |
| **Downregulated in IOPD** |  |  |  |  |
| SUCLG2 | Succinyl-CoA ligase [GDP-forming] subunit beta | Energy metabolism  Mitochondrial | -1.98 | 0.0296 |
| ARL8B | ADP-ribosylation factor-like protein 8B | Lysosomal transport  Vesicle fusion | -1.80 | 0.0366 |
| OXCT1 | Succinyl-CoA:3-ketoacid coenzyme A transferase 1 | Metabolic  mitochondrial | -1.60 | 0.0370 |
| NAPA | Alpha-soluble NSF attachment protein | Synaptic vesicle priming | -1.54 | 0.0023 |
| ALDH1L1 | Cytosolic 10-formyltetrahydrofolate dehydrogenase | Metabolic | -1.53 | 0.0370 |
| HK1 | Hexokinase-1 | Metabolic | -1.42 | 0.0309 |
| STOML2 | Stomatin-like protein 2 | Inter organelle communication  Mitochondrial | -1.35 | 0.0414 |
| FTH1 | Ferritin heavy chain | Metabolic | -1.18 | 0.0279 |
| SDHB | Succinate dehydrogenase [ubiquinone] iron-sulfur subunit, | Metabolic  Mitochondrial | -1.15 | 0.0008 |
| EPRS | Bifunctional glutamate/proline--tRNA ligase;Glutamate--tRNA ligase;Proline--tRNA ligase | Downstream effector in mTORC1 signalling pathway | -1.14 | 0.0254 |
| **Upregulated in IOPD** |  |  |  |  |
| PTGES3 | Prostaglandin E synthase 3 | Metabolic | 1.01 | 0.0436 |
| LMCD1 | LIM and cysteine-rich domains protein 1 | Positive regulator for muscle mass | 1.03 | 0.0315 |
| SMPX | Small muscular protein | Muscle repair  Striated muscle contraction | 1.06 | 0.0058 |
| RAD23A | UV excision repair protein RAD23 homolog A | Catabolic process  Protesomal degradation | 1.07 | 0.0138 |
| TRDN | Triadin | Triad junction  Muscle contraction | 1.21 | 0.0167 |
| AMPD1 | AMP deaminase 1 | Energy metabolism | 1.25 | 0.0410 |
| UQCRQ | Cytochrome b-c1 complex subunit 8 | Energy metabolism  Mitochondrial | 1.37 | 0.0321 |
| ETFDH | Electron transfer flavoprotein-ubiquinone oxidoreductase, | Energy metabolism  Mitochondrial | 1.43 | 0.0474 |
| UBE2N | Ubiquitin-conjugating enzyme E2 N | Proteosome degradation  Negative regulation of TORC1 signalling | 1.57 | 0.0421 |
| TMEM38A | Trimeric intracellular cation channel type A | Muscle contraction | 1.86 | 0.0012 |
| CALM2 | Calmodulin | Muscle contraction | 1.97 | 0.0233 |
| NDUFB3 | NADH dehydrogenase [ubiquinone] 1 beta subcomplex subunit 3 | Energy metabolism  Mitochondrial | 2.03 | 0.0298 |
| COL1A2 | Collagen alpha-2(I) chain | Extracellular Matrix | 2.29 | 0.0498 |
| DDT | D-dopachrome decarboxylase | Inhibits cell growth by regulating TOR pathway | 2.35 | 0.0026 |
| ACTC1 | Actin, alpha cardiac muscle 1 | Sarcomere | 2.63 | 0.0443 |
| HBG2 | Hemoglobin subunit gamma-2 | Catabolic process | 4.52 | 0.0134 |

**Supplementary Table 5:** DAPs in LOPD compared to age matched controls

| **Protein** | **Name** | **Function**  **(selected)** | **log2 Fold-change** | **Adjusted p-Value** |
| --- | --- | --- | --- | --- |
| **Downregulated in LOPD** |  |  |  |  |
| MYL5 | Myosin light chain 5 | Sarcomere | -3.55 | 0.0139 |
| COL3A1 | Collagen alpha-1(III) chain | Extracellular Matrix | -2.76 | 0.0218 |
| TALDO1 | Transaldolase | Energy Metabolism | -2.69 | 0.0299 |
| MT-ATP6 | ATP synthase subunit a | Energy Metabolism  Mitochondrial | -2.05 | 0.0270 |
| USMG5 | Up-regulated during skeletal muscle growth protein 5 | Energy Metabolism  Mitochondrial | -2.02 | 0.0027 |
| CSRP3 | Cysteine and glycine-rich protein 3 | Developmental | -1.94 | 0.0369 |
| MYL6B | Myosin light chain 6B | Sarcomere | -1.90 | 0.0257 |
| MYBPC1 | Myosin-binding protein C | Sarcomere  Slow type | -1.87 | 0.0122 |
| DB1 | Acyl-CoA-binding protein | Transport | -1.86 | 0.0146 |
| TPT1 | Translationally-controlled tumor protein | Protein Biosynthesis | -1.77 | 0.0134 |
| MYH1 | Myosin-1 | Sarcomere  Fast type | -1.73 | 0.0462 |
| SH3BGR | SH3 domain-binding glutamic acid-rich protein | Protein Biosynthesis | -1.69 | 0.0060 |
| TNNI1 | Troponin I | Sarcomere  Thin filament  Slow type | -1.67 | 0.0242 |
| MYBPC2 | Myosin-binding protein C. fast-type | Sarcomere  Fast type | -1.67 | 0.0286 |
| ENO2 | Enolase 2 | Metabolic  Glycolysis | -1.65 | 0.0061 |
| ACYP2 | Acylphosphatase-2 | Metabolic  Mitochondrial | -1.60 | 0.0055 |
| MYOZ1 | Myozenin-1 | Sarcomere  Linking Z-Band proteins | -1.56 | 0.0294 |
| HIST2H2BE | Histone H2B Type 2 E | Maintenance | -1.53 | 0.0436 |
| FKBP3 | **Peptidyl-prolyl cis-trans isomerase** | Immune | -1.50 | 0.0023 |
| MYH4 | Myosin-4 | Sarcomere  Fast type 2B | -1.49 | 0.0272 |
| TPM2 | Tropomyosin beta chain | Sarcomere  Troponin Complex | -1.48 | 0.0132 |
| TNNC1 | Troponin C | Sarcomere  Slow fiber | -1.48 | 0.0205 |
| COL1A2 | Collagen alpha-2(I) chain | Extracellular Matrix | -1.45 | 0.0346 |
| CISD1 | **CDGSH iron-sulfur domain-containing protein 1** | Transport | -1.43 | 0.0176 |
| ST13 | **Hsc70-interacting protein** | Maintenance | -1.42 | 0.0070 |
| TPM3 | Tropomyosin alpha-3 chain | Sarcomere  Troponin Complex | -1.38 | 0.0225 |
| MYL1 | **Myosin light chain 1/3** | Sarcomere | -1.36 | 0.0127 |
| TNNI2 | Troponin I | Sarcomere  Fast fiber | -1.36 | 0.0131 |
| PDLIM3 | PDZ and LIM domain protein 3 | Sarcomere  Actin filament | -1.34 | 0.0160 |
| ATP5D | **ATP synthase subunit delta** | Energy Metabolism  Mitochondrial | -1.30 | 0.0183 |
| MYLPF | Myosin regulating light chain 11 | Sarcomere | -1.22 | 0.0453 |
| NEB | Nebulin | Sarcomere | -1.18 | 0.0262 |
| MYH2 | Myosin-2 | Sarcomere  Fast fiber | -1.16 | 0.0439 |
| FHL3 | Four and a half LIM domains protein 3 | Sarcomere  Actin | -1.16 | 0.0189 |
| PDLIM5 | PDZ and LIM domain protein 5 | Sarcomere  Z-Disk | -1.15 | 0.0113 |
| TPM1 | Tropomyosin alpha-1 chain | Sarcomere | -1.13 | 0.0270 |
| ACTA1 | Actin alpha 1 | Sarcomere  Actin filament | -1.11 | 0.0030 |
| STBD1 | **Starch-binding domain-containing protein 1** | Transport  Glycophagy | -1.11 | 0.0066 |
| ALDH5A1 | **Succinate-semialdehyde dehydrogenase** | Metabolic  Mitochondrial | -1.10 | 0.0038 |
| CHCHD3 | MICOS complex subunit MIC19 | Energy Metabolism  Mitochondrial | -1.07 | 0.0313 |
| SAR1B | **Succinate-semialdehyde dehydrogenase** | Transport | -1.05 | 0.0353 |
| RPS2 | 40S ribosomal protein S2 | Protein Biosynthesis | -1.01 | 0.0300 |
| TRDN | Triadin | Sarcomere  Triad junction | -1.01 | 0.0036 |
| **Upregulated in LOPD** |  |  |  |  |
| MYL4 | Myosin light chain 4 | Sarcomere | 1.00 | 0.0185 |
| HK1 | Hexokinase-1 | Metabolic  Glycolysis | 1.02 | 0.0227 |
| ECl2 | Enoyl-CoA delta isomerase 2 | Energy metabolism  Mitochondrial | 1.02 | 0.0375 |
| PDXK | Pyridoxal kinase | Metabolic | 1.02 | 0.0095 |
| GBE1 | 1.4-alpha-glucan-branching enzyme | Metabolic Glycogen biosynthesis | 1.07 | 0.0163 |
| PSMC1 | 26S protease regulatory subunit 4 | Maintenance | 1.18 | 0.0374 |
| NIT2 | Omega-amidase NIT2 | Metabolic | 1.23 | 0.0025 |
| DYNC1H1 | Cytoplasmic dynein 1 heavy chain 1 | Maintenance | 1.26 | 0.0398 |
| ENDOG | Endonuclease G | Energy metabolism  Mitochondrial | 1.27 | 0.0053 |
| TNXB | Tenascin-X | Extracellular Matrix | 1.31 | 0.0192 |
| PSMB3 | Proteasome subunit beta type-3 | Maintenance | 1.33 | 0.0137 |
| LAP3 | Cytosol aminopeptidase | Metabolic | 1.35 | 0.0090 |
| IDH3B | Isocitrate dehydrogenase [NAD] subunit. mitochondrial; socitrate dehydrogenase [NAD] subunit beta. mitochondrial | Energy metabolism  Mitochondrial | 1.54 | 0.0318 |
| OXCT1 | Succinyl-CoA:3-ketoacid coenzyme A transferase 1 | Energy metabolism  Mitochondrial | 1.64 | 0.0231 |
| GSTK1 | Glutathione S-transferase kappa 1 | Metabolic | 1.54 | 0.0318 |
| PTGES2 | Prostaglandin E synthase 2 | Metabolic | 1.64 | 0.0231 |
| BGN | Biglycan | Extracellular Matrix | 1.54 | 0.0318 |
| ADH1B | Alcohol dehydrogenase 1B | Metabolic | 1.64 | 0.0231 |
| RAB7A | Ras-related protein Rab-7a | Maintenance | 1.54 | 0.0318 |
| CTSD | Cathepsin D | Metabolic | 1.74 | 0.0059 |
| XIRP2 | Xin actin-binding repeat-containing protein 2 | Sarcomere  Actin binding  Muscle lesions  Fast fiber | 1.79 | 0.0210 |
| NES | Nestin | Sarcomere | 1.93 | 0.0063 |
| MVP | Major vault protein | Protein Biosynthesis | 2.08 | 0.0016 |
| VAT1 | Synaptic vesicle membrane protein VAT-1 homolog | Energy Metabolism  Mitochondrial | 2.23 | 0.0003 |

**Supplementary Table 6:** DAPs in treated IOPD (ERT) compared to age matched controls

| **Protein** | **Name** | **Function** | **log2 Fold-change** | **Adjusted p-Value** |
| --- | --- | --- | --- | --- |
| **Downregulated in treated IOPD** |  |  |  |  |
| ZAK | Mitogen-activated protein kinase kinase kinase MLT | Catalytic | -1.58 | 0.0475 |
| SUN2 | SUN domain-containing protein 2 | Cytoskeleton | -1.18 | 0.0406 |
| NDUFS8 | NADH dehydrogenase [ubiquinone] iron-sulfur protein 8 | Energy metabolism  Mitochondrial | -1.08 | 0.0200 |
| **Upregulated in treated IOPD** |  |  |  |  |
| TRDN | Triadin | Cytoskeleton | 1.05 | 0.0358 |
| XPO1 | Exportin-1 | Transport | 1.28 | 0.0346 |
| PIN1 | Peptidyl-prolyl cis-trans isomerase NIMA-interacting 1 | Cell proliferation | 1.39 | 0.0220 |
| PSMB2 | Proteasome subunit beta type-2 | Proteosomal degradation  Protein control  Ubiquitin debendent | 1.41 | 0.0449 |
| PPP2CA | Serine/threonine-protein phosphatase 2A catalytic subunit alpha isoform | Catalytic | 1.42 | 0.0224 |
| QARS | Glutamine--tRNA ligase | Catalytic | 1.46 | 0.0154 |
| PSMD3 | 26S proteasome non-ATPase regulatory subunit 3 | Proteosomal degradation  Protein control  Ubiquitin debendent | 1.54 | 0.0418 |
| PGD | 6-phosphogluconate dehydrogenase, decarboxylating | Metabolic | 1.80 | 0.0333 |
| HMGCS2 | Hydroxymethylglutaryl-CoA synthase | Energy metabolism  Mitochondrial | 1.84 | 0.0417 |
| RPS15 | 40S ribosomal protein S15 | Protein synthesis,  Developmental | 1.84 | 0.0042 |
| MAP2K1 | Dual specificity mitogen-activated protein kinase kinase 1 | Developmental | 1.92 | 0.0054 |
| HSD17B12 | Very-long-chain 3-oxoacyl-CoA reductase | Metabolic  Lipid metabolism | 1.99 | 0.0128 |
| RPN2 | Dolichyl-diphosphooligosaccharide--protein glycosyltransferase subunit 2 | Metabolic  Protein glycosylation | 2.15 | 0.0263 |
| A1BG | Alpha-1B-glycoprotein | Immune regulation | 2.34 | 0.0137 |
| BANF1 | Barrier-to-autointegration factor; Barrier-to-autointegration factor, N-terminally processed | Developmental  Chromatin reorganization | 2.43 | 0.0189 |
| USMG5 | Up-regulated during skeletal muscle growth protein 5  ATPMK | Energy metabolism  Mitochondrial | 3.12 | 0.0032 |
| CALM2 | Calmodulin-2 | Cytoskleton | 3.74 | 0.0016 |
| HBG2 | Hemoglobin subunit gamma-2 | Oxygen transport | 4.01 | 0.0188 |

**Supplementary Table 7:** DAPs in IOPD treated (ERT) compared to naive IOPD

| **Protein** | **Name** | **Function** | **log2 Fold-change** | **Adjusted p-Value** |
| --- | --- | --- | --- | --- |
| **Downregulated in treated IOPD** |  |  |  |  |
| MT-ND1 | NADH-ubiquinone oxidoreductase chain 1 | Energy metabolism  Mitochondrial | -2.19 | 0.0016 |
| NDUFB3 | NADH dehydrogenase [ubiquinone] 1 beta subcomplex subunit 3 | Energy metabolism  Mitochondrial | -1.97 | 0.0318 |
| DCTN2 | Dynactin subunit 2 | Spindle cell organization during mitosis  Developemental | -1.57 | 0.0447 |
| **Upregulated in treated IOPD** |  |  |  |  |
| COPS5 | COP9 signalosome complex subunit 5 | Developmental | 1.27 | 0.0161 |
| COQ5 | 2-methoxy-6-polyprenyl-1,4-benzoquinol methylase, | Metabolic  Mitochondrial | 1.29 | 0.0323 |
| HPX | Hemopexin | Metabolic  Antioxidant | 1.34 | 0.0182 |
| PCBP2 | Poly(rC)-binding protein 2; Poly(rC)-binding protein 3 | DNA/RNA binding  Immune system processes | 1.35 | 0.0379 |
| MAP2K1 | Dual specificity mitogen-activated protein kinase kinase 1 | Developmental | 1.46 | 0.0290 |
| RPS15 | 40S ribosomal protein S15 | Protein synthesis,  Developmental | 1.54 | 0.0252 |
| NDUFB11 | NADH dehydrogenase [ubiquinone] 1 beta subcomplex subunit 11, mitochondrial | Energy metabolism  Mitochondrial | 1.54 | 0.0133 |
| HNRNPA0 | Heterogeneous nuclear ribonucleoprotein A0 | Inflammatory response | 1.55 | 0.0296 |
| GLRX | Glutaredoxin-1 | Catalytic  Antioxidant defense system | 1.83 | 0.0426 |
| HP | Haptoglobin; Haptoglobin alpha chain; Haptoglobin beta chain | IL-6 dependent, Marker for muscle improvement | 1.95 | 0.0048 |
| SGCB | Beta-sarcoglycan | Cytoskeleton | 2.94 | 0.0278 |

**Supplemental Table 8:** Clinical and genetic findings

| **Patient ID** | **Group** | **Gender** | **Age at biopsy** | **Allele 1** | **Allele 2** | **Biopsy site** | **Pompe score** |
| --- | --- | --- | --- | --- | --- | --- | --- |
| I1 | IOPD | m | 0.3 | c.delExon 2-4 | c.del Exon 2-4 | MVL | n.a. |
| I2 | IOPD | m | 0.3 | c.925G>A | c.1927G>A | MVL | n.a. |
| I3a | IOPD | f | 0.1 | c.1933 G > A | c.794delG | MVL | 2,1 |
| I3b | IOPD_ERT | f | 3.5 | c.1933 G > A | c.794delG | MVL | n.a. |
| I4a | IOPD | f | 0.2 | c.104 T > C | c.266 C > A | MVL | 2,2 |
| I4b | IOPD_ERT | f | 2.8 | c.104 T > C | c.266 C > A | MVL | n.a. |
| I5 | IOPD | m | 0.5 | c.307T>G | c.307T>G | MVL | n.a |
| I6 | IOPD | m | 0.4 | c.1456 G > C | c.1456 G > C | MVL | 1,7 |
| I7 | IOPD | m | 0.1 | c.2078dupA | c.2078dupA | MVL | n.a. |
| I8 | IOPD | f | 0.6 | c.525delT | c.1143_114insTAG | MVL | n.a. |
| I9 | IOPD | f | 0.4 | c.525delT | c.1721T>C | MVL | n.a. |
| I10a | IOPD | m | 0.6 | c.2078ins A | c.2078insA | MVL | 3,5 |
| I10b | IOPD_ERT | m | 5.8 | c.2078ins A | c.2078insA | MVL | n.a. |
| I10c | IOPD_ERT | m | 7.3 | c.2078ins A | c.2078insA | MVL | n.a. |
| I11 | IOPD | m | 0.8 | c.2213 G > A | c.2213 G > A | MVL | 4,0 |
| I12 | IOPD_ERT | f | 1.6 | n.k. | n.k. | MVL | n.a. |
| I13 | IOPD_ERT | m | 2.3 | n.k. | n.k. | MVL | n.a. |
| L1 | LOPD | f | 47.0 | c.-32-13T>G | c.2261dupC | MD | n.a. |
| L2 | LOPD | m | 61.0 | c.[-32-13T>G] | c.1127delGinsc | MVL | 0.15 |
| L3 | LOPD | m | 49.0 | c.[-32-13T>G] | c.118C>T | MD | 0.14 |
| L4 | LOPD | m | 50.0 | c.[-32-13T>G] | c.118C>T | MVL | 0.19 |
| L5 | LOPD | m | 65.0 | c.[-32-13T>G] | c.655G>A | MVL | 0.53 |
| L6 | LOPD | f | 40.0 | c.[-32-13T>G | c.271delG | MVL | 0.93 |
| L7 | LOPD | f | 38.0 | c.[-32-13T>G] | c.2242G>T | MBF | 0.77 |
| L8 | LOPD | m | 50.0 | c.[-32-13T>G] | c.1655T>C | MVL | 0.68 |
| L9 | LOPD | f | 51.0 | c.[-32-13T>G] | c.525delT | MVL | 2.23 |
| L10 | LOPD | m | 46.0 | c.[-32-13T>G] | c.307T>G | MBB | 0.38 |
| L11 | LOPD | m | 37.0 | c.[-32-13T>G] | c.794delG | MBB | 0.8 |
| L12 | LOPD | m | 26.0 | c.[-32-13T>G] | c.794delG | MBB | 0.08 |
| L13 | LOPD | f | 31.0 | c.[-32-13T>G] | c.2078_insA | MBB | 1.71 |
| L14 | LOPD | f | 34.0 | c.1655T>C | c.1478C>T | MTA | 1.57 |
| L15 | LOPD | f | 35.0 | c.[-32-13T>G] | c.1655T>C | MBB | 0.52 |
| L16 | LOPD | m | 78.0 | c.[-32-13T>G] | c.925G>A | MVL | 1.02 |
| L17 | LOPD | f | 62.0 | c.[-32-13T>G] | c.525delT | MVL | 0.23 |
| L18 | LOPD | m | 50.0 | n.k. | n.k. | MBF | 0.4 |
| L19 | LOPD | m | 31.0 | c.[-32-13T>G] | c.1495T>A | MVL | 0.51 |
| L20 | LOPD | f | 55.0 | 536bpDel | c.[-32-13T>G] | MD | 0.71 |
| L21 | LOPD | m | 58.0 | c.[-32-13T>G] | c.525delT | MVL | 0.94 |
| L22 | LOPD | m | 30.0 | n..k | n.k. | MVL | n.a. |
| L23 | LOPD | m | 58.0 | n.k. | n.k. | MVL | n.a. |

n.a. = not analyzed. n.k. not know (genetic and muscle morphology was consistent with Pompe disease);

**Supplementary Figures**

**Supplementary Figure 1. Differences of gene and protein regulation in LOPD and IOPD compared to age-matched controls**

Principal component analysis (PCA) of the transcriptomic (all genes) and proteomic data (DAPs). Each dot resembles one muscle sample. PCA revealed distinct groups of IOPD compared to controls and LOPD compared to controls indicating differences in gene and protein expression. Differences in IOPD compared to infantile controls were stronger as LOPD compared to adult controls. In LOPD some overlap of LOPD samples with controls indicates Pompe samples with minor pathology. DAP = Differential abundant protein

**Supplementary Figure 2. Immunofluorescence studies of VAPB and RPS9 expression in disease progression**

Immunofluorescence study and corresponding H&E stained sections from one control and three LOPD samples with low and moderate muscle pathology. VAPB expression is low in healthy muscle and increased in vacuolated muscle fiber. VAPB expression is increased in single altered muscle fibers with punctae in hypertrophic fibers and circumscribed with in section adjacent to basophilic inclusions identified in H&E. Co-expression of RPS9 and fast skeletal myosin MHC2a isoform indicates expression of RPS9 in fast fibers in control, whereas no co-expression of RPS9 with slow skeletal myosin MHC1 isoform is present. The expression of RPS9 is increased in LOPD samples with higher degree of pathology effecting both fiber types (scale bar 50 μm).

**Supplementary Figure 3. Transcriptomic and proteomic deregulation of muscle development and differentiation in IOPD samples compared to controls.**

Pathway analysis reveals downregulation of genes associated with GO term clusters “muscle cell development”, “muscle system process” and "cell-cell signaling by wnt” (a). Heatmap demonstrate downregulated DAPs involved in muscle regeneration, regulation of the calcium balance and contractility (b). Electron microscopy of an IOPD muscle cross-section (left) shows severe disturbance of the myofibrillar architecture with loss of sarcomere structures, extra-lysosomal glycogen (G) and mitochondrial aggregates (MA). The longitudinal section (right) shows increased number of intermyofibrillar mitochondria with increased variance in size and shape (#) (c).

**Supplementary Figure 4. Immunofluorescence studies of BAG3 expression in LOPD disease progression**

Immunofluorescence study and corresponding H&E-stained sections from one control and three LOPD samples with low and moderate muscle pathology. BAG3 is expressed in healthy muscle. BAG3 expression is increased in vacuolated Pompe muscle fibers with disturbance of sarcomeric structure indicated by FilaminC staining and H&E staining. With LAMP2, large protein aggregates are only detected in Pompe muscle fibers (scale bar 50 μm).

**Supplementary Figure 5. Immunofluorescence studies of Galectin-3 expression in LOPD disease progression**

Immunofluorescence study and corresponding H&E-stained section from one control and two LOPD samples with low and moderate muscle pathology. Galectin is not expressed in healthy muscle. Galectin expression is increased in vacuolated Pompe muscle fibers with disturbance of sarcomeric structure indicated by FilaminC and H&E staining (scale bar 50 μm).

**Supplementary Figure 6. Gene Ontology pathway analyses of metabolic regulation in LOPD and IOPD**

Gene Ontology pathway analysis of genes associated with metabolic function in naive Pompe samples compared to controls reveals strong downregulation in LOPD (a) and IOPD samples (b). TEM images of Pompe samples show strong mitochondrial alterations with polymorphous shape and para-crystalline inclusions in LOPD (c) and IOPD (d).

**Supplementary Figure 7. Gene Ontology and Hallmark pathway analyses associated with immune regulation in LOPD and IOPD**

Gene set enrichment analysis (GSEA) using Gene Ontology (GO) (a,d) and Hallmark (b,e) reveal an upregulation of pathways associated with immune regulation in LOPD (a,b) and IOPD (d,e,d) samples. Immunohistochemistry with antibodies against MHC I reveals stronger expression on muscle fiber in LOPD samples with severe pathology compared to samples with minor pathology. With CD3 a few more T-cells can be detected in sever pathology (c).
